# Excess cysteine drives conjugate formation and impairs proliferation of NRF2-activated cancer cells

**DOI:** 10.1038/s42255-026-01499-8

**Published:** 2026-04-07

**Authors:** Jennifer A. Brain, Anna-Lena B. G. Vigil, Kristian Davidsen, Ayaha Itokawa, Abby C. Jurasin, Hannah J. Kerbyson, Maximilian Kobiesa, Madeleine L. Hart, Sang Jun Yoon, Peter Bellotti, Juan Pablo Maianti, Gina M. DeNicola, Lucas B. Sullivan

**Affiliations:** 1 Human Biology Division, Fred Hutchinson Cancer Center, Seattle, WA USA; 2 Department of Metabolism and Physiology, Moffitt Cancer Center, Tampa, FL USA; 3 Chemical Synthesis Core, Department of Biochemistry, Albert Einstein College of Medicine, Bronx, NY USA

**Keywords:** Cancer metabolism, Metabolomics, Metabolism

## Abstract

Cancer cells with constitutive NRF2 activation take up excess cystine beyond the cysteine demands of conventional pathways, implying unknown metabolic fates. Here, we develop an unbiased approach for the identification of cysteine metabolic fates and find that both known and previously uncharacterized cysteine-derived metabolites accumulate in NRF2-activated cancer cells. We identify many of these unknown metabolites as conjugates formed between cysteine and endogenous sugar metabolites, which can also be generated in vitro. We confirm the presence of these cysteine-derived conjugates in murine lung cancer models and primary human lung cancer samples, and their enrichment in NRF2-activated tumours in each context. Mechanistically, NRF2 promotes cystine uptake by driving SLC7A11 expression, which increases intracellular cysteine levels to promote these cysteine fates in a panel of cancer cell lines. Finally, we show that NRF2 activation creates a sensitivity to high environmental cystine, which impairs cell proliferation through excess free cysteine, and can be mitigated by sequestration into cysteine-derived conjugates. Overall, these findings reveal a cancer-associated metabolic vulnerability to excess cysteine stress, and reveal unrecognized routes of cysteine metabolism.

## Abstract (web summary)

Previously uncharacterized cysteine-derived conjugates, including with endogenous sugar metabolites, accumulate in cancer cells with constitutive NRF2 activation and account for some of the increased cystine uptake that cannot be explained by conventional cysteine metabolism.

## Main

Metabolites are fundamental units of cellular systems, supporting cell function through essential roles, including energy generation, macromolecular synthesis, stress defence, signalling and structure. While the major metabolites of mammalian cells have been known for decades, auxiliary molecules specific to cellular contexts likely remain undiscovered^1^. Developing a more complete catalogue of the metabolome is crucial for understanding factors governing human health and particularly cancer, where alterations to metabolism are an inextricable feature of the disease.

While the metabolic state of a cancer is influenced by many variables, including genotype, lineage and environment^2^, the recurring nature of specific genetic changes within and across cancer types results in convergent metabolic phenotypes, termed metabotypes. One such metabotype is driven by activation of the transcription factor NRF2 (encoded by the gene *NFE2L2*), which drives a gene expression programme that modifies cell metabolism, including changes to central carbon metabolism, glutamine catabolism, redox fluxes, mitochondrial function and glutathione synthesis^3–9^. Constitutive NRF2 activation is prevalent in human cancers and models have demonstrated interactions between NRF2 stabilization and tumour initiation, progression and chemoresistance^8,10–15^. The changes associated with the NRF2 metabotype suggests a potential vulnerability to metabolism-targeting therapies, although the identification of efficacious targets remains a challenge.

**Fig. 1:**
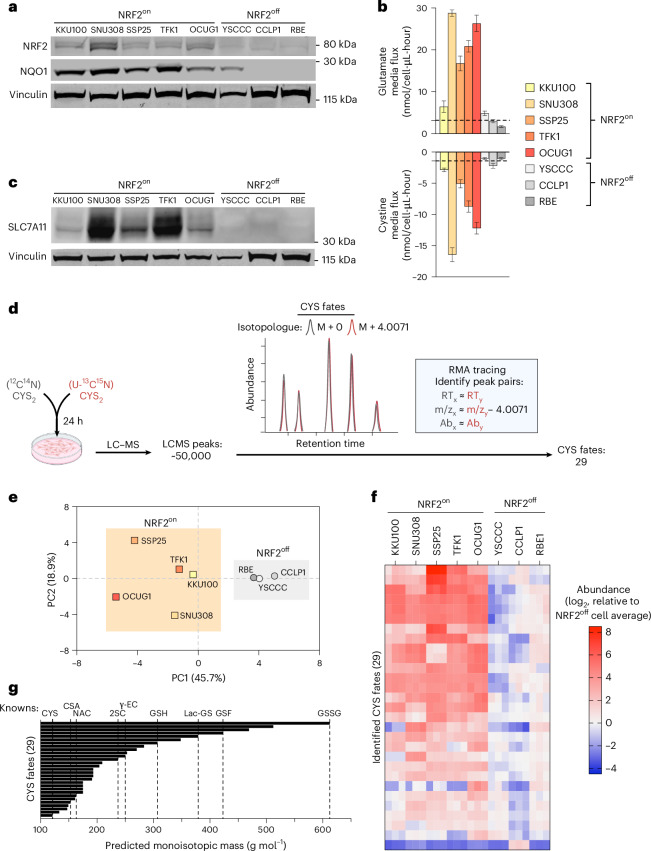
NRF2 activation is associated with increased cystine consumption and accumulation of known and unknown cysteine fates. **a**, Western blot for NRF2, NQO1 and Vinculin for bile duct cancer cell lines designated as NRF2^on^ or NRF2^off^, as indicated. Vinculin is used as a loading control. These results are representative of three separate experiments with similar results. **b**, Metabolite fluxes for medium cystine and glutamate measured by liquid chromatography–mass spectrometry (LC–MS) metabolomics in NRF2^on^ cells and NRF2^off^ bile duct cancer cell lines, as indicated. Rates were determined over multiple time points for each cell line, with *n* = 3 for each time point. Positive values indicate increased concentrations over time (production) and negative values indicate decreased medium concentrations over time (consumption). Bar terminus represents calculated flux value and error bars represent the standard error of the linear regression slope used to calculate metabolite flux. **c**, Western blot for SLC7A11 and Vinculin for NRF2^on^ and NRF2^off^ bile duct cancer cell lines, as indicated. Vinculin is used as a loading control. These results are representative of three separate experiments with similar results. **d**, Schematic depicting how incubation with an equimolar mixture of unlabelled (^12^C^14^N) and fully labelled (U-^13^C^15^N) cystine (CYS_2_) can generate metabolic fates of cysteine (CYS) measurable by LC–MS as peak pairs with near identical retention times and abundances but with a mass shift of 4.0071 (M + 4.0071), corresponding to incorporation of ^13^C_3_^15^N_1_. RMA-tracing algorithm identifies peak pairs with similar retention times (RT), abundances (Ab) and with mass to charge (*m*/*z*) ratios shifted by 4.0071, which identified 29 potential CYS fates in the LC–MS dataset from the bile duct cancer cell line panel. **e**, Principal-component analysis of the variation in abundance of the 29 CYS fates identified by RMA tracing across bile duct cell lines. Clustering of NRF2^on^ (squares) and NRF2^off^ cell lines (circles) are highlighted. **f**, Heatmap of the abundances of CYS fates identified by RMA tracing, ranked by the highest average enrichment in NRF2^on^ cell lines compared with the average abundance across NRF2^off^ cell lines. **g**, Predicted monoisotopic masses of identified CYS fates, annotated with masses of known CYS fates also identified in the same dataset. NAC, *N*-acetylcysteine; 2SC, S-(2-succinyl)-cysteine; γ-EC, γ-glutamylcysteine; Lac-GS, lactoylglutathione; GSF, succinated glutathione (also known as succinicGSH or S(1,2-dicarboxyethyl)glutathione); CSA, cysteine sulfinic acid. Panel **d** created in BioRender; Sullivan, L. https://biorender.com/wtofmpq (2026). Source data

**Extended Data Fig. 1:**
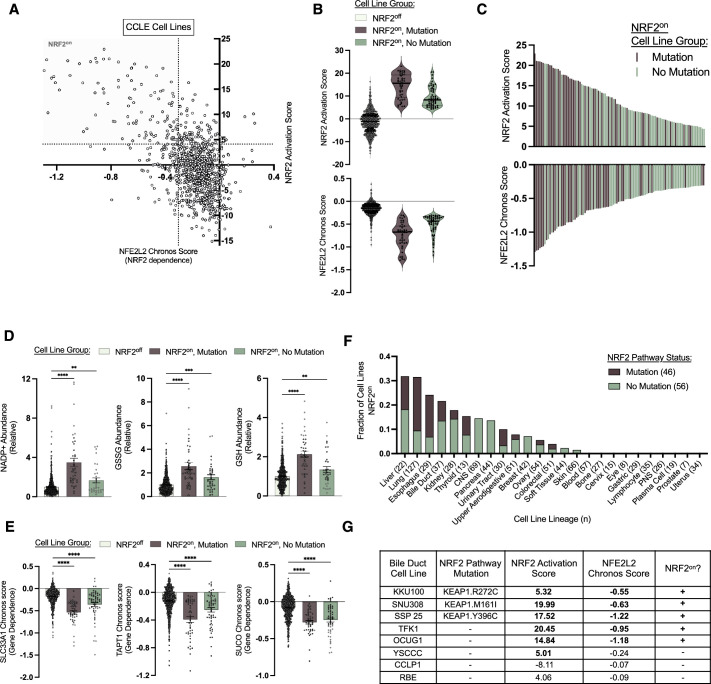
NRF2 activation is prevalent across cancer cell lines and not exclusive to those with canonical activating mutations. (**A**) Scatterplot for the relationship between NRF2 dependence, defined by a negative Chronos Score for *NFE2L2*, and NRF2 activation, calculated based on the variance of mRNA expression of 9 canonical NRF2 target genes, across 973 cancer cell lines from the Cancer Dependency Map. NRF2^on^ cells are depicted in the upper left quadrant formed by the dotted lines, which are those in the highest quintile for both NRF2 dependency and NRF2 activation. Outside of this group are referred to as NRF2^off^ cells. (**B**) Average NRF2 activation score and *NFE2L2* dependency depicted for NRF2^off^ cell lines (*n* = 871) and NRF2^on^ cell lines (*n* = 102, total), splitting NRF2^on^ cells (*n* = 102, total) into those with (*n* = 46) or without (*n* = 56) an annotated, potentially NRF2-activating mutation in *NFE2L2*, *KEAP1*, or *CUL3*. (**C**) Histogram of cell lines ranked by NRF2 activation score (upper) or NRF2 dependency (*NFE2L2* Chronos score) (lower), with the presence or absence of potential NRF2-activating mutations depicted by colour. (**D**) Relative abundance of three metabolites that have been reported to be enriched in cell lines with NRF2 activation (NADP+, glutathione disulfide (GSSG) and glutathione (GSH)), across cell lines that were assigned a NRF2^on^ status (with or without potential NRF2-activating mutations) or NRF2^off^ status, that have available metabolomics measurements. *n* = 626 (NADP+) or *n* = 617 (GSSG and GSH). NADP: NRF2^on^, mutation *P* < 0.0001, NRF2^on^, no mutation *P* = 0.0039. GSSG: NRF2^on^, mutation *P* < 0.0001, NRF2^on^, no mutation *P* = 0.0002. GSH: NRF2^on^, mutation *P* < 0.0001, NRF2^on^, no mutation *P* = 0.0047. (**E**) Average Chronos dependency score for three genes that have been reported to be coessential in NRF2-activated cell lines (*SLC33A1*, *TAPT1*, and *SUCO*), for each cell line group. *n* = 973 total cell lines, with *n* = 871 NRF2^off^ cell lines, *n* = 46 NRF2^on^, mutation, and *n* = 56 NRF2^on^, no mutation. All comparisons *P* < 0.0001. (**F**) Fraction of cell lines for each annotated tissue lineage with NRF2^on^ status, with the presence or absence of potential NRF2-activating mutations depicted by colour. Numbers in x-axis labels (n) refer to the total number of cell lines analysed from that lineage, with a minimum of seven cell lines from a lineage being required for graphing. (**G**) NRF2 activation scores and *NFE2L2* Chronos scores for the eight bile duct cancer cell lines used in this study, with 5 assigned NRF2^on^ status (KKU100, SNU308, SSP25, TFK1, OCUG1) and 3 assigned NRF2^off^ status (YSCCC, CCLP1, RBE). For violin plots (**B**), solid black line is the median and broken grey lines are quartiles. For bar charts (**D**, **E**) error bars are S.E.M. Statistical significance was assessed using one-way ANOVA with Dunnett’s correction for multiple comparisons (**D**, **E**). ***P < 0.001, ****P < 0.0001. Source data

To evaluate cancer cell lines exhibiting chronic NRF2 activation, we evaluated 913 candidates from the Cancer Dependency Map (https://depmap.org)^16,17^ for cell lines with evidence of both increased NRF2 target gene expression and *NFE2L2* genetic dependency, termed NRF2^on^ cells (Extended Data Fig. 1a). NRF2^on^ status was not exclusive to cell lines with canonical NRF2-activating mutations, and NRF2^on^ cell lines with or without activating mutations had NRF2-associated metabolic changes and gene coessentialities (Extended Data Fig. 1b–e)^18,19^. NRF2^on^ status was enriched in tissue lineages conventionally associated with oncogenic NRF2 activation, including lung and oesophagus, and in lineages with emerging roles for NRF2 activation in tumourigenesis, including cancers from the bile duct (Extended Data Fig. 1f)^20^. We chose to first investigate the metabolic consequences of NRF2 activation within a single lineage, selecting eight bile duct cancer cell lines for characterization: five with NRF2^on^ status and three without constitutive NRF2 activation, termed NRF2^off^ (Extended Data Fig. 1g). This demarcation was confirmed by western blot, as NRF2^on^ cells had increased expression of NRF2 and its conventional transcriptional target NQO1 compared with NRF2^off^ cells (Fig. 1a).

**Extended Data Fig. 2:**
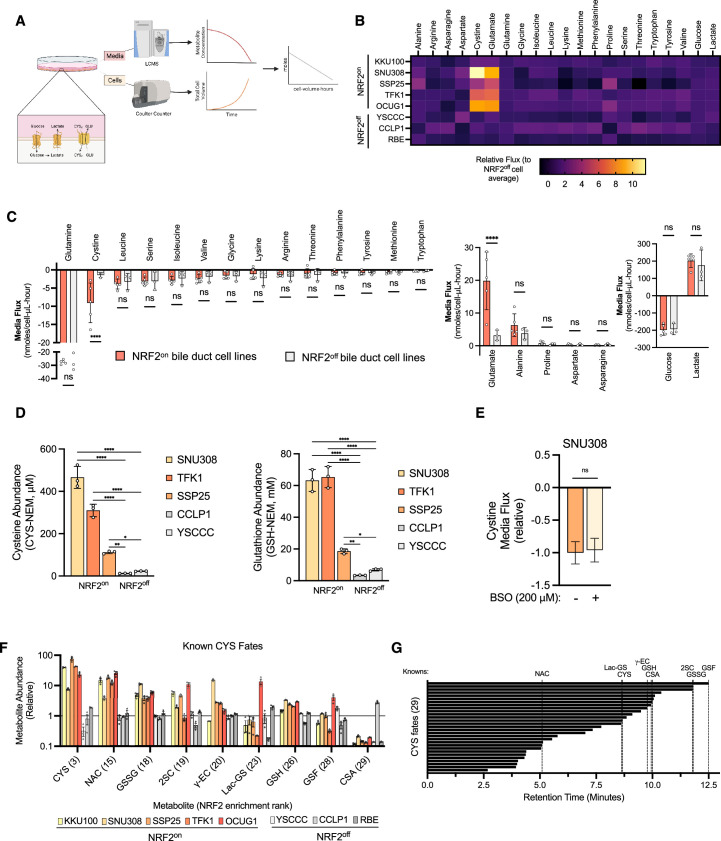
Metabolite consumption rates and measurements of intracellular CYS fates identified by RMA tracing in bile duct cancer cell lines. (**A**) Schematic depicting quantification of media metabolites and cell biomass over time to calculate metabolite fluxes. (**B**) Heatmap of the fold change in consumption rates of metabolites for each cell line, relative to the average value of the three NRF2^off^ cell lines. (**C**) Average media fluxes of individual metabolites, comparing rates between NRF2^on^ cells and NRF2^off^ cells. Left graph consists of amino acids supplied in DMEM, centre graph consists of amino acids not supplied in DMEM, and right graph consists of sugars. Individual fluxes were determined over multiple time points for each cell line, with *n* = 3 for each time point. Grouped rates shown here are the average of consumption rates across the cell lines of each category, *n* = 5 for NRF2^on^ and = 3 for NRF2^off^. Glutamine: *P* > 0.9999. Cystine: *P* < 0.0001. Leucine: *P* > 0.9999. Serine: *P* > 0.9999. Isoleucine: *P* > 0.9999. Valine: *P* > 0.9999. Glycine: *P* > 0.9999. Lysine: *P* = 0.9997. Arginine: *P* > 0.9999. Threonine: *P* > 0.9999. Phenylalanine: *P* > 0.9999. Tyrosine: *P* > 0.9999. Methionine: *P* > 0.9999. Tryptophan: *P* > 0.9999. Glutamate: *P* < 0.0001. Alanine: *P* = 0.8712. Proline: *P* > 0.9999. Aspartate: *P* > 0.9999. Asparagine: *P* > 0.9999. Glucose: *P* = 0.9903. Lactate: *P* = 0.708. (**D**) Absolute quantification of intracellular cysteine, measured as CYS–NEM, or intracellular glutathione, measured as GSH-NEM, in five bile duct cancer cell lines cultured in 200 μM CYS_2_ for 2 h and extracted with NEM and measured by LC–MS. *n* = 3. CYS-NEM: CCLP1 vs SNU308 *P* < 0.0001, CCLP1 vs TFK1 *P* < 0.0001, YSCCC vs SNU308 *P* < 0.0001, YSCCC vs TFK1 *P* < 0.0001, CCLP1 vs SSP25 *P* = 0.0064, YSCCC vs SSP25 *P* = 0.0135. GSH-NEM: CCLP1 vs SNU308 *P* < 0.0001, CCLP1 vs TFK1 *P* < 0.0001, YSCCC vs SNU308 *P* < 0.0001, YSCCC vs TFK1 *P* < 0.0001, CCLP1 vs SSP25 *P* = 0.0099, YSCCC vs SSP25 *P* = 0.0483. (**E**) Relative media flux of cystine in SNU308 cells cultured in 200 μM CYS_2_ with vehicle or 200 μM BSO, relative to the cystine flux of untreated cells for 24 h. Negative value indicates net consumption. *n* = 3. (**F**) Relative metabolite levels across cell lines for each of nine known CYS fates identified by RMA tracing that had been verified by chemical standards (*n* = 3). Relative ion counts are calculated as relative to the average of NRF2^off^ cell average for each metabolite. CYS: cysteine, NAC: N-acetylcysteine, GSSG: glutathione disulfide, 2SC: S-(2-succinyl)-cysteine, γ-EC: γ-glutamylcysteine, Lac-GS: lactoylglutathione, GSH: glutathione, GSF: succinated glutathione (also known as succinicGSH or S(1,2-dicarboxyethyl)glutathione), CSA: cysteine sulfinic acid. Numbers in x-axis labels represent the rank of most enriched CYS metabolites in NRF2^on^ cell lines compared to NRF2^off^ cell lines from RMA tracing, corresponding to the row number in Fig. 1f. *n* = 3. (**G**) Chromatographic retention times of RMA tracing identified 29 CYS fates, annotated with retention times of known CYS fates, related to Fig. 1g. Error bars are SEM. Statistical significance was assessed using two-way ANOVA with Sidak’s correction for multiple comparisons (**C**, **D**) or by unpaired two-tailed Student’s t-test (**E**). ns = not significant, *P < 0.05, **P < 0.01, ****P < 0.0001. Panel **a** created in BioRender; Sullivan, L. https://biorender.com/wtofmpq (2026). Source data

To identify metabolic differences associated with NRF2 activation, we quantified medium metabolic fluxes across our bile duct cancer cell line panel (Extended Data Fig. 2a). While most metabolites fluxes were comparable across both groups, NRF2^on^ cells had increased cystine (CYS_2_) consumption and glutamate efflux compared with the NRF2^off^ cell lines (Extended Data Fig. 2b,c). These results are consistent with findings in lung cancer cells, where NRF2 activation drives cystine uptake via increased expression of SLC7A11, the limiting component of the heterodimeric CYS_2_/glutamate antiporter complex xCT^5,9,21^. Indeed, SLC7A11 expression was higher in NRF2^on^ bile duct cell lines compared with the NRF2^off^ bile duct cell lines and correlated with CYS_2_ consumption rates (Fig. 1b,c). Consistent with higher CYS_2_ uptake, NRF2^on^ lines compared with the NRF2^off^ lines have substantially higher levels of intracellular cysteine (CYS) and glutathione (GSH) (Extended Data Fig. 2d). We considered whether the heightened demands for CYS utilization for GSH synthesis in NRF2^on^ cells was sufficient to explain the increased CYS_2_ consumption, but excluded this possibility when inhibition of the GSH synthesis enzyme glutamate–cysteine ligase (GCL) by buthionine sulfoximine (BSO) treatment did not repress CYS_2_ uptake in NRF2^on^ cells (Extended Data Fig. 2e). As these CYS_2_ consumption changes also occurred without commensurate changes to the consumption of essential amino acids (Extended Data Fig. 2b,c), much of the CYS_2_ consumption in NRF2^on^ cells is likely diverted into metabolic fates other than for its conventional roles in GSH or protein synthesis.

To investigate how NRF2 activation changes CYS_2_ metabolism, we developed an untargeted isotope-tracing approach for unbiased identification of metabolic fates of CYS_2_ using mass spectrometry. This approach took inspiration from other metabolite credentialing methods, where cells are incubated in isotopically labelled nutrients and the subsequent incorporation of heavy atoms into mass spectrometry features signifies that they are metabolic fates deriving from the parent nutrient^22,23^. To identify the metabolic fates of CYS_2_, we incubated cells in medium containing an equimolar mixture of labelled [^13^C_6_,^15^N_2_] CYS_2_ and unlabelled [^12^C_6_,^14^N_2_] CYS_2_, such that the metabolic fates of CYS_2_ incorporate the heavy or light cohort of atoms at approximately equal proportions. As imported CYS_2_ is rapidly reduced to the monomer CYS in cells, the metabolic fates of CYS will appear in mass spectrometry datasets as pairs of peaks with near identical retention times, *m*/*z* values differing only by the incorporation of ^13^C_3_,^15^N (+4.0071), and with similar ion abundances. We cultured our bile duct cell line panel with a mixture of labelled and unlabelled CYS_2_ for 24 h, extracted metabolites, and used untargeted liquid chromatography–mass spectrometry (LC–MS) to generate a peak list of all detected metabolites. We then used a custom designed peak search algorithm, incorporating analyte retention time, *m*/*z* and abundance values, entitled RMA tracing, to find these isotopologue peak pairs, predicted to correspond with the metabolic fates of CYS, ultimately yielding 29 features (Fig. 1d and Methods). Of note, all nine expected CYS fates, verified by chemical standards, were accounted for among the 29 features, highlighting the ability of untargeted RMA tracing to identify authentic metabolic fates (Extended Data Fig. 2f). Principal-component analysis of the abundance of all 29 features across the bile duct cancer cell line panel revealed that cell lines cluster according to NRF2 status, supporting convergent effects on CYS metabolism among the NRF2^on^ metabotype (Fig. 1e). Consistent with increased CYS_2_ consumption, most of these CYS-derived molecules were elevated in abundance in NRF2^on^ cells compared with NRF2^off^ cells (Fig. 1f and Extended Data Fig. 2f). Pending chemical identification, we denoted these mass spectrometry features using the naming convention of ‘C’, for CYS fate, followed by monoisotopic neutral mass and chromatographic retention time (for example, C193_7.3). At this stage it seemed unlikely that all these features ascribed as unknown CYS fates derived from mass spectrometry artifacts^24^, as they cover a range of predicted monoisotopic masses and retention times (Fig. 1g and Extended Data Fig. 2g).

**Fig. 2:**
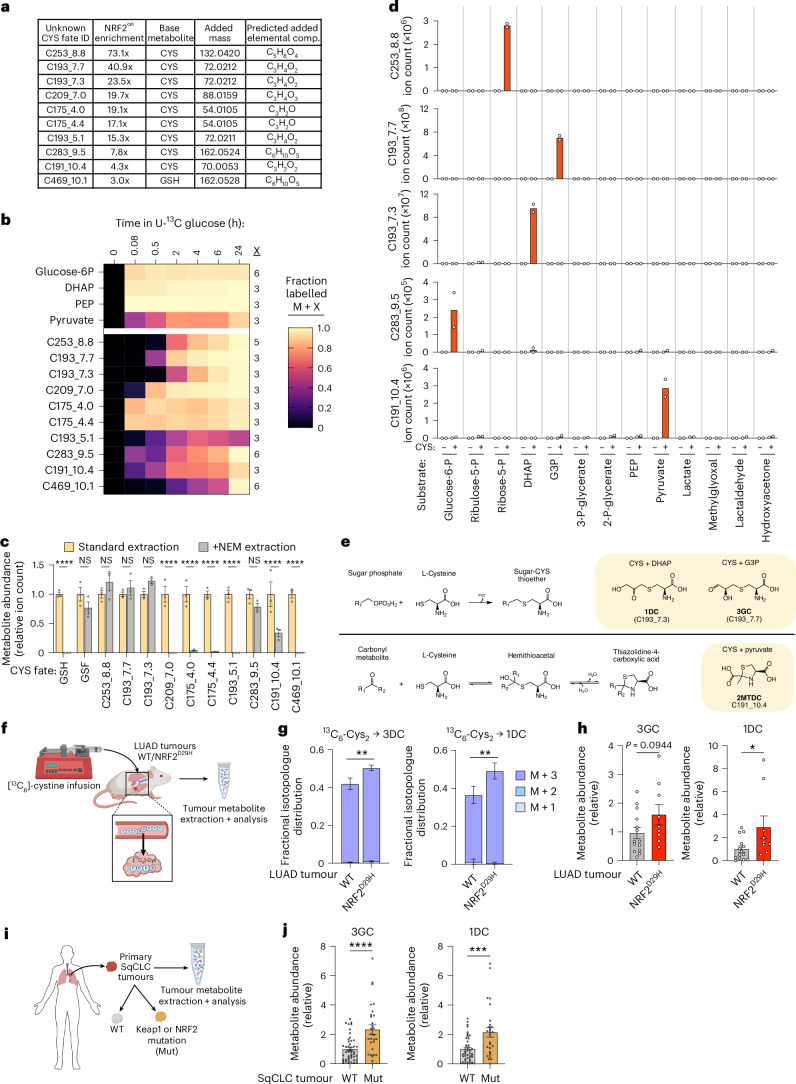
Cysteine reacts with glucose-derived metabolites to generate metabolites enriched in NRF2-activated cells and tumours. **a**, Table describing ten unknown CYS fates with sugar-like added masses, each annotated by ‘C,’ the predicted neutral molecular weight integer, and the retention time. Each includes values for the average abundance enrichment across NRF2^on^ cell lines compared with NRF2^off^ cell lines (NRF2^on^ enrichment), the base metabolite, the implied added mass (by subtracting the monoisotopic exact mass of the base metabolite), and the resulting predicted elemental composition of the added mass. **b**, Fractional isotopologue enrichment of glycolytic intermediates glucose-6-phosphate (glucose-6P), DHAP, phosphoenolpyruvate (PEP), pyruvate and unknown CYS fates as measured by LC–MS metabolomics after culturing SSP25 cells in [U-^13^C] glucose containing medium for the indicated times. For each analyte the dominant isotopologue is shown, which corresponds to all carbons for glycolytic intermediates or the number of carbons in the predicted added elemental composition for unknown CYS fates. Mean value for *n* = 3 per time point is shown. Full isotopologue distributions can be found in Extended Data Fig. 3e,f. **c**, Relative abundance of known and unknown CYS fates measured by LC–MS metabolomics from SSP25 cells extracted in standard conditions or in extraction solvent containing the thiol-reactive conjugating agent *N*-ethylmaleimide (NEM). *n* = 3 replicate wells per condition. GSH, C209_7.0, C175_4.0, C175_4.4, C193_5.1, C191_10.4, C469_10.1: *P* < 0.0001, GSF *P* = 0.5401, C253_8.8 *P* = 0.7037, C193_7.7 *P* = 0.9959, C193_7.3 *P* = 0.5822 and C283_9.5 *P* = 0.6516. **d**, LC–MS measurements of C253_8.8, C193_7.7, C193_7.3, C283_9.5 or C191_10.4 after purified chemical standards of glucose metabolic fates (substrates) were incubated with or without cysteine for 1 h at 37 °C. *n* = 2 separate reactions. **e**, Schematic of two proposed groups of identified CYS fates, deriving from reactions between CYS and sugar phosphates to generate stable sugar–CYS thioether conjugates or CYS and carbonyl-containing molecules to generate reversible hemithioacetal and thiazolidine-4-carboxylic acid products. **f**, Schematic depicting the ^13^C_6_-CYS_2_ 4-h infusion in autochthonous murine LUAD tumours without (WT) or with activation of a genetically encoded mutant of NRF2 (NRF2^D29H^) and subsequent LC–MS metabolomics. **g**, Mass isotopologue fraction of labelled species (M + 1-M + 3) of sugar–CYS conjugates extracted from LUAD murine tumours as depicted in **f**. 3GC: WT, *n* = 16 tumours, NRF2^D29H^, *n* = 10 tumours, M + 3: *P* = 0.004. 1DC: WT, *n* = 11 tumours, NRF2^D29H^, *n* = 10 tumours, M + 3: *P* = 0.0017. **h**, Relative ion count (total of all isotopologues) for sugar–CYS conjugates measured by LC–MS metabolomics. Relative abundance was calculated to the average of the WT group for each metabolite. *n* = 10, 1DC: *P* = 0.0356. **i**, Schematic depicting the collection and LC–MS metabolomics of primary human SqCLC samples without (WT) or with annotated KEAP1 or NRF2 mutations (Mut). **j**, Relative ion count for sugar–CYS conjugates in primary SqCLC tumours measured by LC–MS metabolomics. 3GC: WT, *n* = 47 tumours, Mut, *n* = 29 tumours, *P* < 0.0001. 1DC: WT, *n* = 44 tumours, Mut, *n* = 29 tumours, *P* = 0.0004. Error bars show s.e.m. (**c**,**d**,**g**,**h**,**j**). Statistical significance was assessed using two-way analysis of variance (ANOVA) with Sidak’s correction for multiple comparisons (**c**,**g**) or two-tailed unpaired Student’s t-test (**h**,**j**). For **g**, highlighted comparison is for M + 3 species, whereas comparisons of M + 1 and M + 2 were not significant. NS, not significant, *P < 0.05, **P < 0.01, ***P < 0.001, ****P < 0.0001. Panels **f** and **i** created in BioRender; Brain, J. https://biorender.com/wtofmpq (2026). Source data

**Extended Data Fig. 3:**
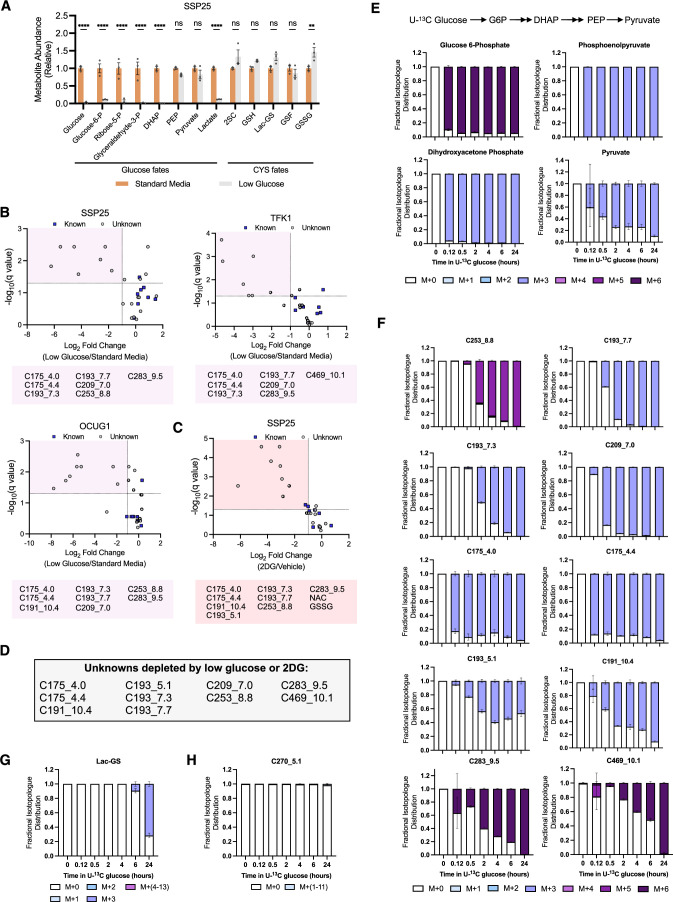
Characterization of glycolysis metabolites and cysteine fates that incorporate carbons from glucose. (**A**) Relative abundances of glucose fates and CYS fates measured by LC–MS metabolomics from SSP25 cells cultured in standard DMEM media (25 mM glucose) or in low glucose DMEM (250 μM glucose) for 6 h. Relative abundance was calculated to average ion count of standard media group for each metabolite. *n* = 3 replicate wells per condition. Glucose, Glucose-6-P, Ribose-5-P, Glyceraldehyde-3-P, DHAP, Lactate *P* < 0.0001. PEP *P* = 0.8290. Pyruvate *P* = 0.8974. 2SC *P* = 0.1109. GSH *P* = 0.6809. Lac-GS *P* = 0.1586. GSF = 0.9795. GSSG *P* = 0.0058. (**B**) Volcano plots of differential CYS fate abundances from NRF2^on^ cell lines (SSP25, OCUG1, and TFK1). Each point represents a CYS fate. X-axis denotes the log_2_ fold change of CYS fates as measured by LC–MS metabolomics between cells cultured in standard DMEM media (25 mM glucose) or in low glucose DMEM (250 μM glucose) for 6 h. Y-axis shows the negative log_10_-transformed q-value derived from multiple unpaired t tests. Features with a log_2_ fold change of < -1 and adjusted q-value < 0.05 (FDR correction) are highlighted and listed below each graph. *n* = 3 replicate wells per condition. (**C**) Volcano plot of differential CYS fate abundances from SSP25 cells cultured in vehicle or 5 mM 2-deoxyglucose (2DG) for 6 h. Each point represents a CYS fate. X-axis denotes the log_2_ fold change of CYS fates as measured by LC–MS metabolomics between cells Y-axis shows the negative log_10_-transformed q-value derived from multiple unpaired t tests. Features with a fold change < -1 and adjusted q-value < 0.05 (FDR correction) are highlighted and listed below the graph. *n* = 3 replicate wells per condition. (**D**) Summarized table of unknown CYS fates depleted in low glucose or upon treatment with 2DG. (**E**) Fractional isotopologue distribution of glycolytic intermediates glucose-6-phosphate (G6P), dihydroxyacetone phosphate, phosphoenolpyruvate, and pyruvate as measured by LC–MS metabolomics after culturing SSP25 cells in media containing [U-^13^C] glucose for the indicated times. *n* = 3 replicate wells per condition. (**F**) Fractional isotopologue distribution of 10 unknown CYS fates with sugar-like added masses as measured by LC–MS metabolomics after culturing SSP25 cells in media containing [U-^13^C] glucose for the indicated times. *n* = 3 replicate wells per condition. (**G**) Fractional isotopologue distribution of lactoylglutathione (Lac-GS) as measured by LC–MS metabolomics after culturing SSP25 cells in media containing [U-^13^C] glucose for the indicated times. *n* = 3 replicate wells per condition. (**H**) Fractional isotopologue distribution of CYS conjugate C270_5.1, a fate not predicted to derive carbon from glucose, as measured by LC–MS metabolomics after culturing SSP25 cells in media containing [U-^13^C] glucose for the indicated times. *n* = 3 replicate wells per condition. Error bars are SEM. *n* = 3. Statistical significance was assessed using two-way ANOVA with Sidak’s correction for multiple comparisons (A). ns = not significant, **P < 0.01, ****P < 0.0001. Source data

To gain chemical information about unknown CYS fates we next used the calculated exact mass of each identified fate to predict its elemental composition and its implied added mass (subtracting the mass of CYS or GSH, as appropriate), using biologically relevant atoms and heuristics of plausible molecular formulas^25^. We noted that ten of the detected CYS fates, including those among the most enriched in NRF2^on^ cells, had implied added masses corresponding to sugar-like elemental compositions, suggesting that these fates may arise from the addition of sugar-derived atoms to CYS (Fig. 2a and Supplementary Table 1). In tissue culture settings intracellular sugars primarily derive from glucose; so we tested whether disruptions to glucose metabolism could impact the abundance of CYS fates in three NRF2^on^ cell lines. Culturing cells in low-glucose conditions resulted in expected depletions to metabolites in glycolysis and the pentose phosphate pathway and also depleted the unknown CYS fates with sugar-like added masses (Extended Data Fig. 3a,b). Treatment with the glycolysis inhibitor 2-deoxyglucose (2DG) similarly depleted these unknown metabolites (Extended Data Fig. 3c). Notably, all ten CYS fates with sugar-like added masses were depleted in at least one glucose metabolism impairing condition (Extended Data Fig. 3d). As these treatments did not substantially deplete other known or unknown CYS fates (Extended Data Fig. 3a–d), we concluded that these effects were specific to the engagement of CYS with sugar metabolism rather than indirect effects of impairing glucose utilization on CYS metabolism. Overall, these results indicate that interactions between glucose metabolism and CYS are a convergent feature among several unknown CYS fates.

To test directly whether the unknown CYS-derived metabolites incorporate carbon from glucose, we cultured cells in medium containing [U-^13^C] glucose and measured the isotope incorporation into metabolites over time. As expected, glycolytic intermediates rapidly incorporate ^13^C from glucose, with mass shifts (M + X) corresponding to the number of carbons that they derive from glucose (X) (Fig. 2b and Extended Data Fig. 3e). Of note, the CYS fates with sugar-like added masses all incorporated ^13^C from glucose, with each displaying mass shifts corresponding to their predicted added mass elemental composition (Fig. 2a,b and Extended Data Fig. 3f). These labelling patterns are mirrored by lactoyl-GSH, a known CYS fate that incorporates carbon from glucose, and are absent in other CYS fates that were not predicted to derive carbon from glucose (Extended Data Fig. 3g,h). Notably, the delayed kinetics of isotope enrichment in CYS fates from [U-^13^C] glucose compared with glycolytic intermediates demonstrates that their formation cannot be explained by chemical reactions occurring during metabolite extraction, and that they are instead authentic ‘sugar–CYS’ metabolic fates (Fig. 2b and Extended Data Fig. 3e–h).

**Extended Data Fig. 4:**
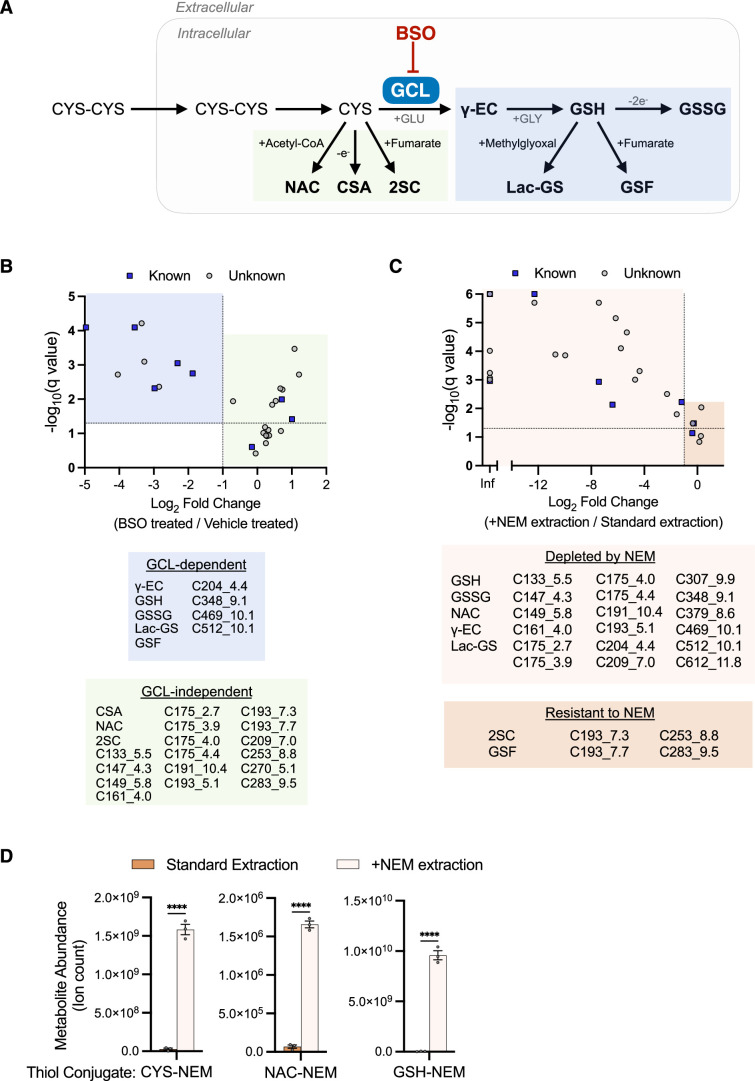
Effects of glutathione synthesis inhibition or extraction with the conjugating agent NEM on cysteine fate abundances. (**A**) Schematic of cysteine metabolism and glutathione (GSH) synthesis, highlighting metabolic branch points of each. Buthionine sulfoximine (BSO) is an inhibitor of the glutathione synthesis enzyme glutamate-cysteine ligase (GCL). (**B**) Volcano plot showing differential CYS fate abundances. Each point represents a CYS fate. X-axis denotes the log_2_ fold change of CYS fates as measured by LC–MS metabolomics between SSP25 cells cultured in 200 μM CYS_2_ for 24 h with vehicle or 50 μM BSO. Y-axis shows the negative log10-transformed q-value derived from multiple unpaired t tests. Features with at least a two-fold depletion by BSO and an adjusted q-value < 0.05 (FDR correction) are highlighted in blue as ‘GCL-dependent’. Features that do not meet these criteria are highlighted in green as ‘GCL-independent’. *n* = 3 replicate wells per condition. (**C**) Volcano plot of differential CYS fate abundances from SSP25 cells with standard extraction (80% MeOH) or NEM extraction (80% MeOH with 2 mM ammonium formate in water, pH 7 and 0.5 mM N-methylmaleimide). Each point represents a CYS fate. X-axis denotes the log_2_ fold change of CYS fates as measured by LC–MS metabolomics between cells. Y-axis shows the negative log_10_-transformed q-value derived from multiple unpaired t tests. Samples with a log_2_ fold change labelled ‘Inf’, were undetectable in +NEM extraction conditions. Features with a fold change < −1 and adjusted q-value < 0.05 (FDR correction) are highlighted in the lighter shade as ‘Depleted by NEM’. Features that do not meet these criteria are highlighted the darker shade as ‘Resistant to NEM’. *n* = 3 replicate wells per condition. (**D**) Ion counts measured by LC–MS metabolomics from the same dataset as in (**C**) for three NEM-conjugated variants of the thiol-containing parent metabolites cysteine (CYS-NEM, *P* < 0.0001), N-acetylcysteine (NAC-NEM, *P* < 0.0001), and glutathione (GSH-NEM, *P* < 0.0001). Error bars are SEM. *n* = 3 replicate wells per condition. Statistical significance was assessed using unpaired two-tailed Student’s t-test (**D**). ****P < 0.0001. Source data

As GSH is abundant in NRF2-activated cells, we also tested whether the origin of these CYS fates could arise as downstream byproducts of GSH conjugates. To evaluate this possibility, we treated the NRF2^on^ cell line SSP25 with BSO and measured its effects on the abundance of detected CYS fates. For the known CYS fates, BSO depleted GCL-dependent CYS fates, but not GCL-independent CYS fates, as expected (Extended Data Fig. 4a,b). Notably, among the 20 unknown CYS fates only four were substantially depleted by BSO treatment, indicating that the majority derive from CYS independent of GSH metabolism (Extended Data Fig. 4b and Supplementary Table 1).

A differentiating chemical feature of CYS is its thiol group, which can serve as a nucleophile in spontaneous and/or enzyme-assisted chemical reactions. In some cases, such as a Michael addition, CYS reactions generate thioethers that are effectively irreversible (conjugates), whereas other CYS products, such as the generation of hemithioacetals or 4-carboxy-thiazolidines, can exist in a reversible equilibrium^26–32^. Thus, CYS fates can be stratified by thiol status, where some molecules are without a chemically reactive thiol due to irreversible conjugation (for example GSF^33^) and others maintain a chemically reactive thiol, either because the added mass occurs on non-thiol functional group(s) (for example as in GSH synthesis) or because the thiol is only reversibly occupied. To identify which of these groups each CYS fate corresponds to, we extracted SSP25 cells with the thiol conjugating agent *N*-ethylmaleimide (NEM) to irreversibly react with (and thereby deplete) molecules with chemically available thiols. This approach was validated by the depletion of control CYS fates with available thiols and detection of their conversion into their NEM conjugates (Fig. 2c and Extended Data Fig. 4c,d). Notably, only four unknown CYS fates were found to be resistant to depletion from NEM conjugation, highlighting that multiple chemical processes contribute to the full suite of unknown CYS fates, which can be bifurcated into those with or without chemically available thiols (Extended Data Fig. 4c and Supplementary Table 1).

**Extended Data Fig. 5:**
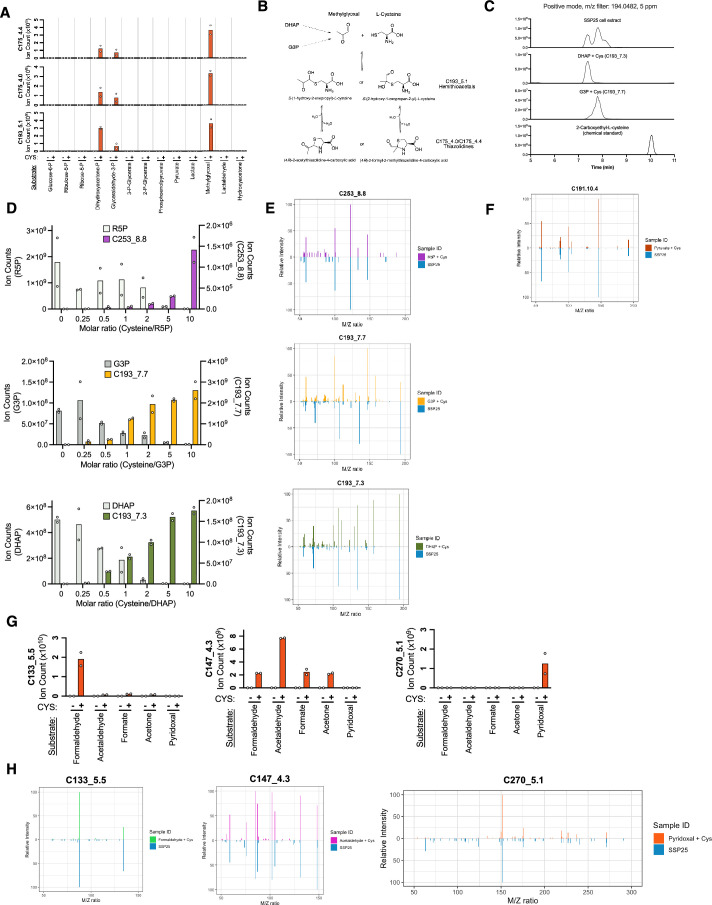
Characterization of identified CYS fates. (**A**) LC–MS metabolomics ion counts of three hypothesized methylglyoxal-derived CYS fates after purified chemical standards of glucose metabolic fates (substrates) were incubated with or without purified cysteine for one hour at 37 **°**C. *n* = 2 separate reactions. (**B**) Proposed chemical structures of hemithioacetal and thiazolidine fates of the reaction between cysteine and methylglyoxal. (**C**) LC–MS chromatography for isobaric peaks from samples of SSP25 cell extract compared to the product of a cell-free reaction of combining CYS with either dihydroxyacetone phosphate (DHAP) or glyceraldehyde-3-phosphate (G3P) or to a chemical standard of 2-carboxyethyl-L-cysteine. (**D**) Ion counts measured by LC–MS metabolomics for three sugar-CYS fates and their parent sugar phosphate compounds, from cell-free reaction systems combining increasing molar ratios of CYS with each sugar phosphate. *n* = 2. (**E**) Tandem mass spectrometry (MS/MS) fragmentation patterns for the three CYS fates generated in a cell-free system by combining sugar phosphates with CYS compared to the corresponding analytes extracted from SSP25 cells. (**F**) MS/MS fragmentation patterns for the CYS fate, C191_10.4, generated in a cell-free system by comining pyruvate with CYS compared to the corresponding analyte extracted from SSP25 cells. (**G**) LC–MS metabolomics ion counts of three hypothesized carbonyl-derived CYS fates after purified chemical standards (substrates) were incubated with or without purified cysteine for one hour. *n* = 2. (**H**) MS/MS fragmentation patterns for the three CYS fates generated in a cell-free system by combining the annotated carbonyl metabolites with CYS compared to the corresponding molecules extracted from SSP25 cells. Source data

Given the inherent reactivity of the CYS thiol group^26–28,30,33,34^, we hypothesized that combining CYS with purified glucose-derived metabolites could generate some of the sugar–CYS metabolites non-enzymatically. Indeed, upon combining CYS with glucose-derived metabolites we were able to generate eight of the sugar–CYS fates, with five being generated only by combining CYS with a single sugar metabolite (Fig. 2d). The combination of CYS with methylglyoxal generated three CYS fates, all of which were also detected to a lesser degree in samples incubated in dihydroxyacetone phosphate (DHAP) and G3P, molecules known to decompose spontaneously into methylglyoxal (Extended Data Fig. 5a,b)^30,35^. Matching the observations in SSP25 cell extracts, the isobaric C193 molecules arising from DHAP and G3P with CYS formed chromatographically separate peaks, which were distinct from 2-carboxyethyl-L-cysteine, another isobaric metabolite found predominantly in plants (Extended Data Fig. 5c)^36^. In addition, combining CYS with sugar phosphates at different molar ratios caused dose-dependent production of their corresponding CYS conjugates (Extended Data Fig. 5d). These synthesized CYS fates also showed similar MS/MS fragmentation patterns to their associated ions from SSP25 cell extracts, further verifying their shared identity (Extended Data Fig. 5e).

Among the sugar–CYS metabolites identified by RMA tracing, our results stratify these molecules into two major groups. The first group consists of products that are not depleted by NEM treatment and are formed from a reaction between CYS and a sugar phosphate (C253_8.8, C193_7.7, C193_7.3 and C283_9.5). We propose these products to result from the CYS thiol undergoing a nucleophilic attack on a sugar phosphate, with the phosphate serving as a leaving group, generating a functionally irreversible thioether ‘sugar–CYS conjugate’ (Fig. 2e). The second group consists of compounds that are at least partially depletable by NEM treatment, in which we propose the CYS thiol reversibly reacts with carbonyls on sugar molecules to generate hemithioacetal or thiazolidine compounds. One notable example is the sugar–CYS thiazolidine compound 2-methyl-2,4-thiazolidine dicarboxylate (2MTDC), which forms from combining CYS and pyruvate and corresponds to C191_10.4 (Fig. 2d,e and Extended Data Fig. 5f)^26,28^. This finding led us to consider that other carbonyl-containing metabolites may also generate other reversible CYS fates, and so we screened reactions between CYS and several biologically relevant carbonyl molecules to determine if they could similarly generate RMA-tracing hits. Indeed, three additional unknown CYS fates were generated from CYS reacting with formaldehyde, acetaldehyde or pyridoxal, all of which had MS/MS fragmentation patterns that matched their associated ions from cell extracts (Extended Data Fig. 5g,h)^27,31,37,38^.

To better understand the biology influencing the production of unknown CYS fates, we focused on the two irreversible sugar–CYS conjugates deriving from G3P and DHAP (C193_7.7 and C193_7.3) as they were among the best detected, most enriched in NRF2^on^ cells, and were stable in diverse extraction conditions. To corroborate the site of CYS thiol reactivity on these 3-carbon sugar metabolites, we reacted CYS with synthetic precursors with alternative leaving groups to phosphate, yielding chemical mixtures with similar LC–MS features as C193_7.7 and C193_7.3 (Supplementary Information Fig. 1). Following the naming convention used for other cysteine conjugates, we will hereafter refer to these conjugates as *S*-(3-(3-deoxy)-glyceraldehyl)–cysteine: 3GC (C193_7.7) and *S*-(1-(1-deoxy)-dihydroxyacetonyl)–cysteine: 1DC (C193_7.3) (Fig. 2e).

**Extended Data Fig. 6:**
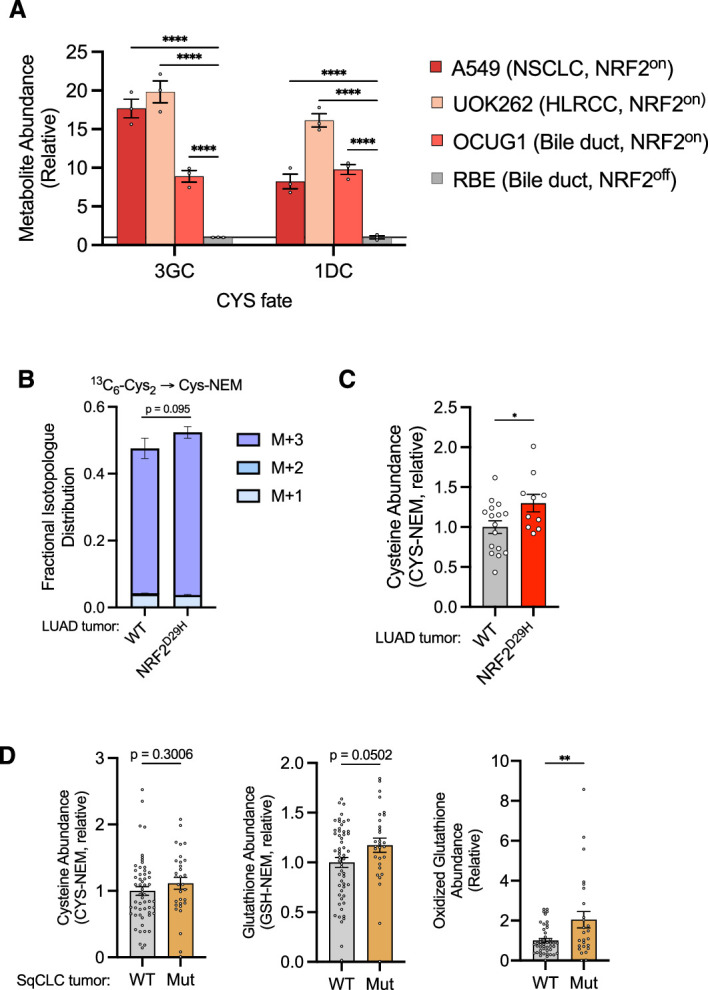
NRF2 activation is associated with increased CYS metabolism across cell lines and tumours. (**A**) Relative abundances of sugar-CYS conjugates in the NRF2^off^ bile duct cell line RBE compared to NRF2-activated and dependent cell lines from other tissues, including the KEAP1-mutant non-small cell lung cancer (NSCLC) cell line A549 (NRF2 activation score; 19.14, *NFE2L2* Chronos Score; -0.65) and the fumarate hydratase (FH) mutant hereditary leiomyomatosis renal cell carcinoma (HLRCC) cell line UOK262 (Not present in depmap database, NRF2 activation and dependence observed in references^33,39^), and the NRF2^on^ bile duct cell line OCUG1. *n* = 3 replicate wells per condition. All comparisons *P* < 0.0001. (**B**) Mass isotopologue fraction of CYS, measured as CYS-NEM, (M + 1-M + 3) in murine LUAD tumours with or without expression of NRF2^D29H^ as measured by [U-^13^C] tracing, extraction with NEM, and LC–MS metabolomics. *n* = 16 tumours (WT) *n* = 10 tumours (NRF2^D29H^). (**C**) Relative abundance of cysteine, detected as CYS-NEM, measured by LC–MS metabolomics between murine LUAD tumours with or without expression of NRF2^D29H^. Abundances are relative ion counts to the average of the WT group. *n* = 16 tumours (WT) *n* = 10 tumours (NRF2^D29H^). *P* = 0.0343. (**D**) Relative abundance of three known cysteine fates, with cysteine measured as CYS-NEM and glutathione measured as GSH-NEM and oxidized glutathione, as measured by LC–MS metabolomics in primary squamous cell carcinoma (SqCLC) tumours with (Mut) or without (WT) a mutation associated with NRF2 activation. CYS-NEM and GSH-NEM: WT, *n* = 56 tumours, Mut, *n* = 31 tumours. GSSG: WT, *n* = 46 tumours, Mut, *n* = 26 tumours, *P* = 0.0027. Error bars are SEM. Statistical significance was assessed using two-way ANOVA with Sidak’s correction for multiple comparisons (**A**) or by unpaired two-tailed Student’s t-test (**B**, **C**, **D**). *P < 0.05, **P < 0.01, ***P < 0.001, ****P < 0.0001. Source data

We next asked whether NRF2^on^ status and increased sugar–CYS metabolism is generalizable to other tissue lineages and physiological contexts. Indeed, in two additional NRF2^on^ cell lines, the KEAP1-mutant non-small cell lung cancer (NSCLC) cell line A549 and the fumarate hydratase mutant, hereditary leiomyomatosis renal cell carcinoma cell line UOK262, both had sugar–CYS conjugate levels comparable with the NRF2^on^ bile duct cell OCUG1 (Extended Data Fig. 6a)^8,33,39^. We next considered how NRF2 status impacts sugar–CYS metabolism in cancers within physiological settings. We measured sugar–CYS conjugates in tumour metabolite extracts from autochthonous murine lung adenocarcinoma (LUAD) tumours initiated by expression of Kras^G12D^ and p53 loss, with or without expression of an activating mutant of NRF2 (NRF2^D29H^), from mice that had been infused with ^13^C_6_-CYS_2_ for 4 h (ref. ^7^) (Fig. 2f). Consistent with increased sugar–CYS metabolism, NRF2^D29H^ tumour extracts displayed increased fractional labelling of M + 3 3GC and 1DC compared with tumours with wild-type (WT) NRF2 (Fig. 2g). NRF2^D29H^ tumours also had a greater abundance of 3GC and 1DC compared with WT tumours (Fig. 2h). CYS levels were also increased in NRF2^D29H^ tumours without statistically different M + 3 labelling (Extended Data Fig. 6c). While the segregation of tumour genotypes by sugar–CYS conjugate levels was modest compared with some cell culture comparisons, we note that NRF2^D29H^ tumours in this model partially suppress NRF2 expression and activity during late-stage disease, potentially obscuring group separation^14,40^.

We next investigated sugar–CYS conjugates in the context of human cancer by LC–MS from primary human squamous cell lung cancer (SqCLC) samples. Samples were separated into those without NRF2-activating mutations (WT) or those annotated with putative NRF2-activating mutations to *NFE2L2* or *KEAP1* (Mut) (Fig. 2i)^41^. Of note, tumours with NRF2-activating mutations had greater abundance of both 3GC and 1DC compared with their WT counterparts (Fig. 2j). These tumours were also associated with increased oxidized glutathione, likely reflecting oxidation of a larger GSH pool before NEM addition, and smaller changes to CYS and GSH (Extended Data Fig. 6d). Altogether, these data indicate that these sugar–CYS conjugates are produced in physiological settings and are increased in NRF2-activated tumours.

**Extended Data Fig. 7:**
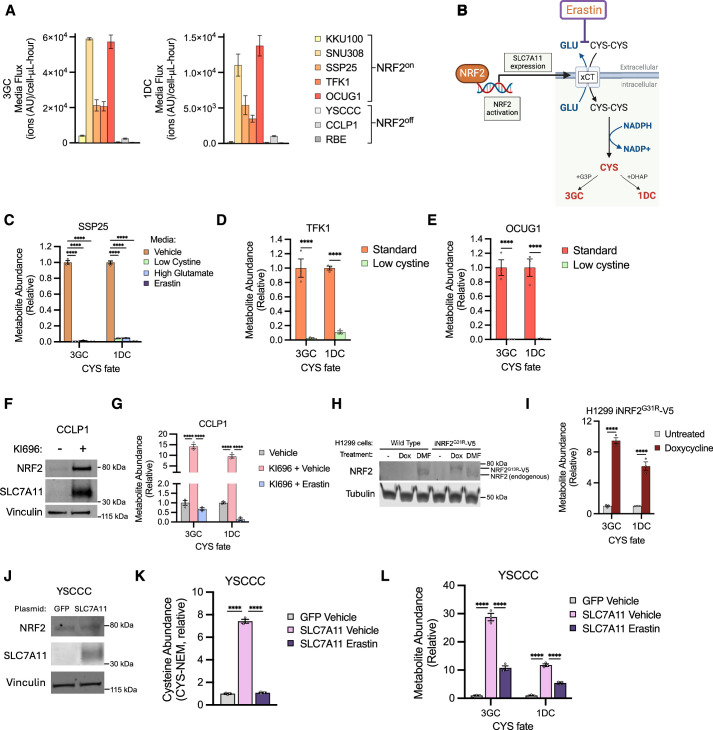
NRF2-driven SLC7A11 expression increases xCT activity, which is sufficient and required for increased production of glucose-derived cysteine conjugates. (**A**) Media fluxes for sugar-CYS conjugates measured by LC–MS metabolomics in NRF2^on^ cells and NRF2^off^ bile duct cancer cell lines, as indicated. Positive values indicate net efflux. Rates were determined over multiple time points for each cell line, with *n* = 3 wells for each time point. Error bars represent the standard error of the linear regression slope used to calculate production rates. (**B**) Schematic depicting NRF2 activation leading to SLC7A11 overexpression and increased xCT activity. CYS_2_ uptake is tied to additional metabolic processes including glutamate (GLU) export and NADPH oxidation, CYS accumulation, and sugar-CYS conjugate formation. Schematic also shows the action of the inhibitor erastin to inhibit xCT. (**C**) Relative abundances of sugar-CYS conjugates measured by LC–MS metabolomics in SSP25 cells cultured in standard media or with media containing three methods of xCT inhibition: low CYS_2_ (20 μM), high GLU (6 mM), or erastin (0.5 μM) for 6 h. Relative abundance is calculated to the average ion counts of the vehicle treated group. *n* = 3 replicate wells per condition. All comparisons *P* < 0.0001. Relative abundance of two sugar-CYS conjugates in the NRF2^on^ cell lines TFK1 (**D**) or OCUG1 (**E**) cultured in media with standard CYS_2_ levels (200 μM) or low CYS_2_ levels (20 μM). *n* = 3 replicate wells per condition. All comparisons *P* < 0.0001. (**F**) Western blot for NRF2, SLC7A11 and Vinculin in CCLP1 cells treated with and without 100 μM KI696, a NRF2 activator, for 24 h. Vinculin is used as a loading control. (**G**) Relative abundance of sugar-CYS conjugates measured by LC–MS metabolomics in CCLP1 cells treated with vehicle control (DMSO) or 100 μM KI696 for 24 h, with additional vehicle control (DMSO) or 0.5 μM erastin for 6 h. Relative abundance is calculated to the average ion count of the vehicle condition for each metabolite. *n* = 3 replicate wells per condition. All comparisons *P* < 0.0001. (**H**) Western blot for NRF2 and Tubulin in the NRF2^off^ NSCLC cell line H1299 (NRF2 activation score; -4.92, *NFE2L2* Chronos Score; -0.08), without alteration (Wild-Type) or in cells ectopically expressing a doxycycline-inducible plasmid for a V5 tagged, activated mutant of NRF2 (NRF2^G31R^-V5) and treated with 1 μg/mL of doxycycline for 6 h or 100 μM dimethyl fumarate (DMF) for 3 h. DMF treatment was used as a control treatment for activation of endogenous NRF2. Tubulin was used as a loading control. (**I**) Relative ion counts of sugar-CYS conjugates in H1299 iNRF2^G31R^-V5 cells treated with and without 1 μg/mL doxycycline for 24 h. Relative abundance is calculated to the average ion count of the untreated condition. *n* = 3 replicate wells per condition. All comparisons *P* < 0.0001. (**J**) Western blot for NRF2, SLC7A11, and Vinculin in YSCCC cells infected with lentivirus expressing eGFP control or SLC7A11. Vinculin is used as a loading control. Relative abundance of cysteine, measured as CYS-NEM, (**K**) or sugar-CYS conjugates (**L**) as measured by LC–MS metabolomics in YSCCC cells ectopically expressing GFP or SLC7A11, with vehicle control (DMSO) or 0.5 μM erastin for 2 h. Abundances are relative ion counts to the average of GFP-expressing cells. *n* = 3 replicate wells per condition. All comparisons *P* < 0.0001. Unless noted, error bars are SEM. Statistical significance was assessed using two-way ANOVA with Sidak’s correction for multiple comparisons (**C**-**E**, **G**, **I**, **K**, **L**). ****P < 0.0001. Panel **b** created in BioRender; Brain, J. & Sullivan, L. https://biorender.com/wtofmpq (2026). Source data

CYS-containing conjugates can be variably excreted from cells, so we considered whether differences in the rate of sugar–CYS metabolite efflux could be a relevant factor for their accumulation. However, the rate of sugar–CYS metabolite export across our bile duct cancer cell line panel was higher in NRF2^on^ cells compared with NRF2^off^ cells, indicating that increased intracellular sugar–CYS metabolite levels must result from increased production rather than decreased excretion (Extended Data Fig. 7a).

These data suggest a simple model by which NRF2 promotes sugar–CYS conjugate levels: NRF2 activation promotes SLC7A11 expression to increase xCT activity, driving CYS_2_ uptake, increasing the intracellular CYS concentration and thereby enabling reactions between CYS and endogenous sugar phosphates (Extended Data Fig. 7b). To determine whether high xCT activity is required for increased levels of sugar–CYS conjugates in NRF2^on^ cells, we impaired its function in SSP25 cells by culturing cells in either low cystine, high glutamate or with the xCT inhibitor erastin. We found that all three treatments decreased sugar–CYS metabolite abundance (Extended Data Fig. 7c). Similar results were also found in low CYS_2_ conditions in other NRF2^on^ cell lines (Extended Data Fig. 7d,e). Thus, high xCT activity is required for increased production of sugar–CYS conjugates in NRF2^on^ cells.

We next probed whether experimental NRF2 activation or SLC7A11 overexpression are sufficient to increase sugar–CYS metabolites. We treated the NRF2^off^ bile duct cancer cell line CCLP1 with KI696, a small-molecule activator of NRF2, which increased NRF2 and SLC7A11 expression, as expected (Extended Data Fig. 7f)^42^. KI696 treatment also correspondingly increased levels of 3GC and 1DC, and this effect was diminished by erastin co-treatment (Extended Data Fig. 7g). Similarly, doxycycline-inducible expression of a degradation-resistant mutant of NRF2 (NRF2^G31R^) in the NRF2^off^ NSCLC cell line H1299 also increased the abundance of sugar–CYS conjugates (Extended Data Fig. 7h,i)^13^. These results demonstrate that NRF2 activation is sufficient to drive the formation of sugar–CYS conjugates in an xCT-dependent manner. Finally, we tested whether increased SLC7A11 expression was sufficient to induce sugar–CYS conjugates in the absence of the broader NRF2 transcriptional programme. Ectopic expression of SLC7A11 in NRF2^off^ YSCCC cells increased SLC7A11 protein levels without affecting NRF2 levels (Extended Data Fig. 7j). SLC7A11 expression also increased levels of intracellular CYS and sugar–CYS conjugates and was inhibited by erastin treatment (Extended Data Fig. 7k,l). Taken together, these results indicate that increased SLC7A11-driven CYS_2_ uptake (via xCT) is the primary mechanism by which NRF2 activation increases the abundance of sugar–CYS conjugates.

**Fig. 3:**
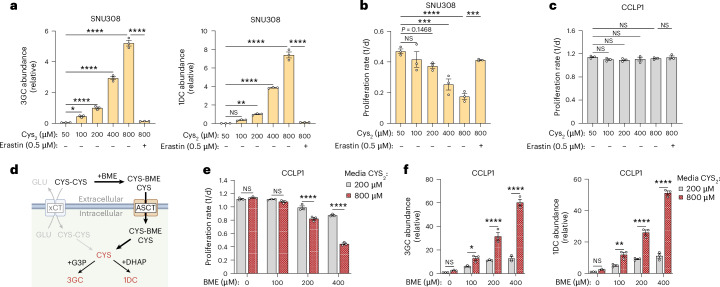
Increased cysteine acquisition causes excess cysteine stress, which increases sugar–CYS conjugates and impairs cancer cell proliferation. **a**, Relative abundance of intracellular sugar–CYS conjugates as measured by LC–MS metabolomics from SNU308 cells upon treatment with different medium concentrations of CYS_2_, with or without 0.5 μM erastin. Abundances are relative ion counts to cells cultured in 200 μM CYS_2_. *n* = 3 replicate wells per condition. 3GC: 50 μM CYS_2_ versus 100 μM CYS_2_ *P* = 0.0498, 50 μM CYS_2_ versus 200 CYS_2_ *P* < 0.0001, 50 μM CYS_2_ versus 400 CYS_2_ *P* < 0.0001, 50 μM CYS_2_ versus 800 μM CYS_2_ *P* < 0.0001, 800 μM CYS_2_ versus 800 μM CYS_2_ + 0.5 μM erastin *P* < 0.0001. 1DC: 50 μM CYS_2_ versus 100 μM CYS_2_ *P* = 0.4228, 50 μM CYS_2_ versus _2_00 μM CYS_2_ *P* = 0.0014, 50 μM CYS_2_ versus 400 CYS_2_ *P* < 0.0001, 50 μM CYS_2_ versus 800 μM CYS_2_ *P* < 0.0001, 800 μM CYS_2_ versus 800 μM CYS_2_ + 0.5 μM erastin *P* < 0.0001. **b**,**c**, Cell proliferation rates of the NRF_2_^on^ cell line SNU308 (**b**) or NRF2^off^ cell line CCLP1 (**c**) treated with different medium concentrations of CYS_2_, with vehicle control (dimethylsulfoxide (DMSO)) or with 0.5 μM erastin. *n* = 3 replicate wells per condition. SNU308: 50 μM CYS_2_ versus 100 μM CYS_2_ *P* = 0.6832, 50 μM CYS_2_ versus 200 μM CYS_2_ *P* = 0.1468, 50 μM CYS_2_ versus 400 μM CYS_2_ *P* = 0.0007, 50 μM CYS_2_ versus 800 μM CYS_2_ *P* < 0.0001, 800 μM CYS_2_ versus 800 μM CYS_2_ + 0.5 μM erastin *P* < 0.0003. **d**, Schematic depicting an xCT-independent route of CYS acquisition, where treatment with β-mercaptoethanol (BME) reacts with medium CYS_2_ to either reduce it to CYS or generate the mixed disulfide, CYS-BME. One or both compounds are imported through the neutral amino acid transporter family (ASCT), resulting in intracellular CYS delivery uncoupled from glutamate export. **e**, Cell proliferation rates of CCLP1 cells treated with different medium concentrations of BME, in medium containing either 200 or 800 μM CYS_2_. *n* = 3 replicate wells per condition, 0 μM BME *P* = 0.8164, 100 μM BME *P* = 0.4121, 200 μM BME *P* = 0.0001, 400 μM BME *P* = 0.0001. **f**, Abundance of intracellular sugar–CYS conjugates as measured by LC–MS metabolomics from CCLP1 cells upon treatment with different medium concentrations of BME, in medium containing either 200 or 800 μM CYS_2_ for 24 h. Abundances are relative ion counts to cells cultured in 200 μM CYS_2_ with 0 μM BME for each metabolite. *n* = 3 replicate wells per condition. 3GC: 0 μM BME *P* = 0.9421, 100 μM BME *P* = 0.0396, 200 μM BME *P* = 0.0001, 400 μM BME *P* = 0.0001. 1DC: 0 μM BME *P* = 0.8724, 100 μM BME *P* = 0.0013, 200 μM BME *P* < 0.0001, 400 μM BME *P* < 0.0001. Error bars show s.e.m. Statistical significance was assessed using one-way ANOVA with Sidak’s correction for multiple comparisons (**a**–**c**) or by two-way ANOVA with Sidak’s correction for multiple comparisons (**e**,**f**). *P < 0.05, **P < 0.01, ***P < 0.001, ****P < 0.0001. Panel **d** created in BioRender; Sullivan, L. https://biorender.com/wtofmpq (2026). Source data

**Extended Data Fig. 8:**
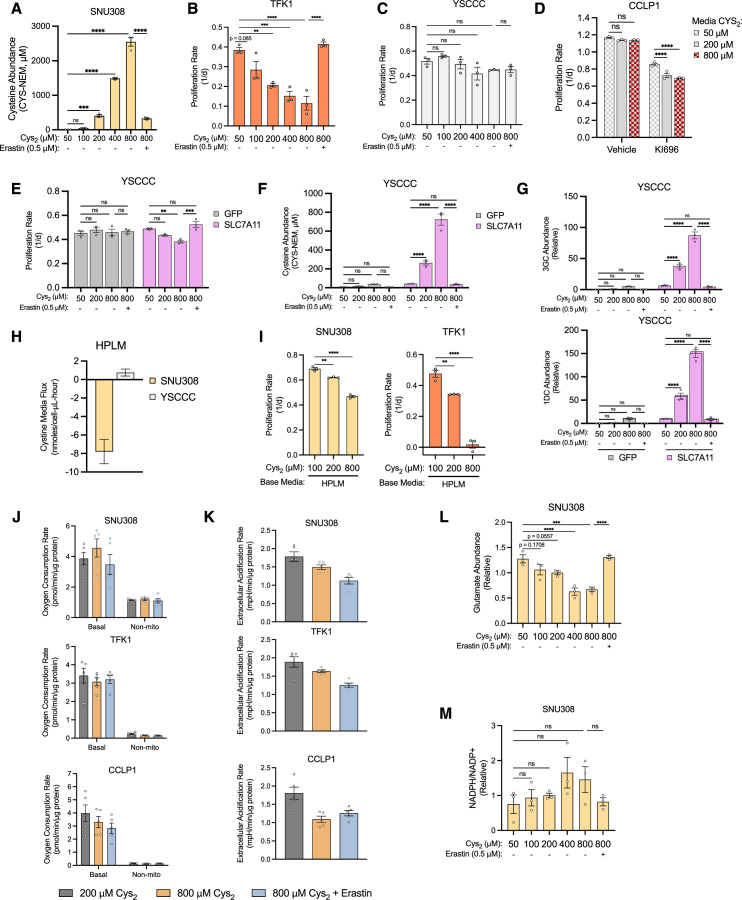
Characterization of metabolic and functional effects of altering xCT activity. (**A**) Absolute quantification of intracellular cysteine, measured as CYS-NEM, in SNU308 cells cultured in media with varying concentrations of CYS_2_ treated with vehicle control (DMSO) or 0.5 μM erastin for 24 h, extracted with NEM, and measured by LC–MS. *n* = 3 replicate wells per condition. 100 μM CYS_2_ *P* = 0.9873, 200 μM CYS_2_ *P* = 0.0009, 400 μM CYS_2_ *P* < 0.0001, 800 μM CYS_2_ *P* < 0.0001, 800 μM CYS_2_ + 0.5 μM erastin *P* < 0.0001. Cell proliferation rates of the NRF2^on^ cell line TFK1 (**B**) or the NRF2^off^ cell line YSCCC (**C**) treated with different media concentrations of CYS_2_, with vehicle control (DMSO) or without 0.5 μM erastin. *n* = 3 replicate wells per condition. TFK1: 100 μM CYS_2_ *P* = 0.0845, 200 μM CYS_2_ *P* = 0.0017, 400 μM CYS_2_ *P* = 0.0002, 800 μM CYS_2_ *P* < 0.0001, 800 μM CYS_2_ + 0.5 μM erastin *P* < 0.0001. (**D**) Cell proliferation rates of CCLP1 cells treated with different media concentrations of CYS_2_, treated with vehicle control (DMSO) or 100 μM KI696. *n* = 3 replicate wells per condition. KI696: all comparisons *P* < 0.0001. (**E**) Cell proliferation rates of YSCCC cells ectopically expressing GFP or SLC7A11 cultured in DMEM without pyruvate with different media concentrations of CYS_2_, and vehicle control (DMSO) or 0.5 μM erastin. *n* = 3 replicate wells per condition. SLC7A11: 50 μM CYS_2_ vs 200 μM CYS_2_ *P* = 0.0040, 800 μM CYS_2_ vs 800 μM CYS_2_ + 0.5 μM erastin *P* = 0.0002. Absolute abundance of cysteine, measured as CYS-NEM (**F**) or relative abundance of sugar-CYS conjugates (**G**) as measured by LC–MS in YSCCC cells ectopically expressing GFP or SLC7A11 cultured in 50 μM, 200 μM, or 800 μM CYS_2_ treated with vehicle control (DMSO) or 0.5 μM erastin for 24 h. Relative abundances were calculated to the average ion count of GFP control in 200 μM, CYS_2_ for each metabolite. *n* = 3 replicate wells per condition, all significant comparisons *P* < 0.0001. (**H**) Fluxes for media cystine of SNU308 and YSCCC cells cultured in human plasma-like media (HPLM) for 24 h. Rates were determined by measuring the change in cystine between time zero (immediately after media change) and 24 h, with samples taken from *n* = 3 replicate wells for each time point. Negative values indicate net consumption. Error bars represent the standard error of the linear regression slope used to calculate consumption rates. (**I**) Cell proliferation rate of NRF2^on^ cell lines SNU308 and TFK1s in HPLM media with varying concentrations of CYS_2_. *n* = 3 replicate wells per condition. SNU308: 100 μM CYS_2_ vs 200 μM CYS_2_ *P* = 0.0013, 100 μM CYS_2_ vs 800 μM CYS_2_ *P* < 0.0001. TFK1: 100 μM CYS_2_ vs 200 μM CYS_2_ *P* = 0.0053, 100 μM CYS_2_ vs 800 μM CYS_2_ *P* < 0.0001. (**J**) Oxygen consumption rates from SNU308, TFK1, and CCLP1 cells at different CYS_2_ concentrations, with or without 0.5 μM erastin treatment for 24 h. Basal indicates the oxygen consumption rate when cells are otherwise untreated while non-mito indicates the residual oxygen consumption rate after treatment with 0.5 μM rotenone and 0.5 μM antimycin. *n* = 5 replicate wells per group. (**K**) Extracellular acidification rate, a surrogate for glycolytic rate, were simultaneously measured in the same conditions as in (**J**). Relative ion count of glutamate (**L**) or relative ion count ratio of NADPH to NADP+ (**M**) as measured by LC–MS metabolomics in SNU308 cells cultured in media with varying concentrations of CYS_2_ treated with vehicle control (DMSO) or 0.5 μM erastin for 24 h. Values are relative to cells cultured 200 μM CYS_2_. *n* = 3 replicate wells per condition. Glutamate: 50 μM CYS_2_ *P* = 0.1708, 200 μM CYS_2_ *P* = 0.0557, 400 μM CYS_2_ *P* < 0.0001, 800 μM CYS_2_ + 0.5 μM erastin *P* < 0.0001, 800 μM CYS_2_ *P* = 0.0001. Error bars are SEM. Statistical significance was assessed using one-way ANOVA with Sidak’s correction for multiple comparisons (**A**-**C**, **I**, **L**, **M**) or two-way ANOVA with Sidak’s correction for multiple comparisons (**D**-**G**). ns = not significant, **P < 0.01, ***P < 0.001, ****P < 0.0001. Source data

The transmembrane concentration gradients of the xCT substrates glutamate and CYS_2_ limits the opportunity for direct feedback regulation, so we considered whether xCT activity might be further determined by the environmental CYS_2_ concentration in cells with high SLC7A11 expression. Supporting this concept, we found that culturing NRF2^on^ cells in variable CYS_2_ resulted in proportionally elevated levels of intracellular cysteine (Extended Data Fig. 8a) and sugar–CYS conjugates (Fig. 3a). The fact that increased xCT activity can substantially increase intracellular CYS abundance and thereby promote reactions with endogenous biomolecules suggests that alterations to CYS levels may impact cell function, so we investigated the functional consequences of increased CYS_2_ uptake on NRF2^on^ bile duct cell lines with high SLC7A11 expression (SNU308 and TFK1) versus NRF2^off^ bile duct cell lines with low SLC7A11 expression (CCLP1 and YSCCC). Both NRF2^on^ cell lines showed dose-dependent cell proliferation impairments according to environmental CYS_2_ levels, whereas both NRF2^off^ cell lines were unaffected by extracellular CYS_2_ levels (Fig. 3b,c and Extended Data Fig. 8b,c). This CYS_2_-driven toxicity was dependent on increased CYS_2_ uptake, as treatment with the xCT inhibitor erastin rescued cell proliferation of NRF2^on^ cells and had no effect on NRF2^off^ cells. Moreover, treatment of NRF2^off^ cell lines with the NRF2 activator KI696 or ectopic expression of SLC7A11 were both sufficient to introduce a modest CYS_2_-dependent toxicity (Extended Data Fig. 8d,e). Similarly, SLC7A11 overexpression in the NRF2^off^ cell line YSCCC also caused CYS_2_-dependent accumulation of intracellular CYS, 3GC and 1DC that was suppressed by erastin treatment, mirroring effects in NRF2^on^ cell lines, (Extended Data Fig. 8f,g). To ensure that these phenotypes are generalizable across medium conditions, we also evaluated them in human plasma-like medium (HPLM)^43^. The NRF2^on^ cell line SNU308 maintained a high CYS_2_ consumption rate in HPLM and both SNU308 and TFK1 cells maintained a CYS_2_ concentration dependent proliferation defect in HPLM (Extended Data Fig. 8h,i). Overall, these results indicate that increased environmental CYS_2_ can drive CYS_2_ uptake via xCT in cells with high SLC7A11 expression to impair cell proliferation.

We next investigated the metabolic mechanisms by which excess CYS_2_ uptake causes toxicity. We evaluated whether high CYS_2_ caused any obvious bioenergetic effects, but did not observe xCT-dependent changes to mitochondrial oxygen consumption or extracellular acidification (Extended Data Fig. 8j,k). CYS_2_ uptake and the conversion to intracellular CYS is also linked to glutamate efflux and NADPH consumption, suggesting an excess of either activity could contribute to toxicity (Extended Data Fig. 7b). While high xCT activity can increase sensitivity to disruptions to glutamate or NADPH regeneration, it is not clear whether these mechanisms impair cell function in the absence of additional metabolic perturbations^4,5,9,11,44–46^. We measured glutamate and NADPH/NADP^+^ in SNU308 cells, finding that increased xCT activity was associated with a decrease in glutamate levels, but not with depletion of NADPH/NADP^+^ (Extended Data Fig. 8l, m). We thus hypothesized that the toxicity of excess xCT-dependent cystine uptake is mediated by intracellular glutamate limitation and/or excess intracellular CYS.

**Extended Data Fig. 9:**
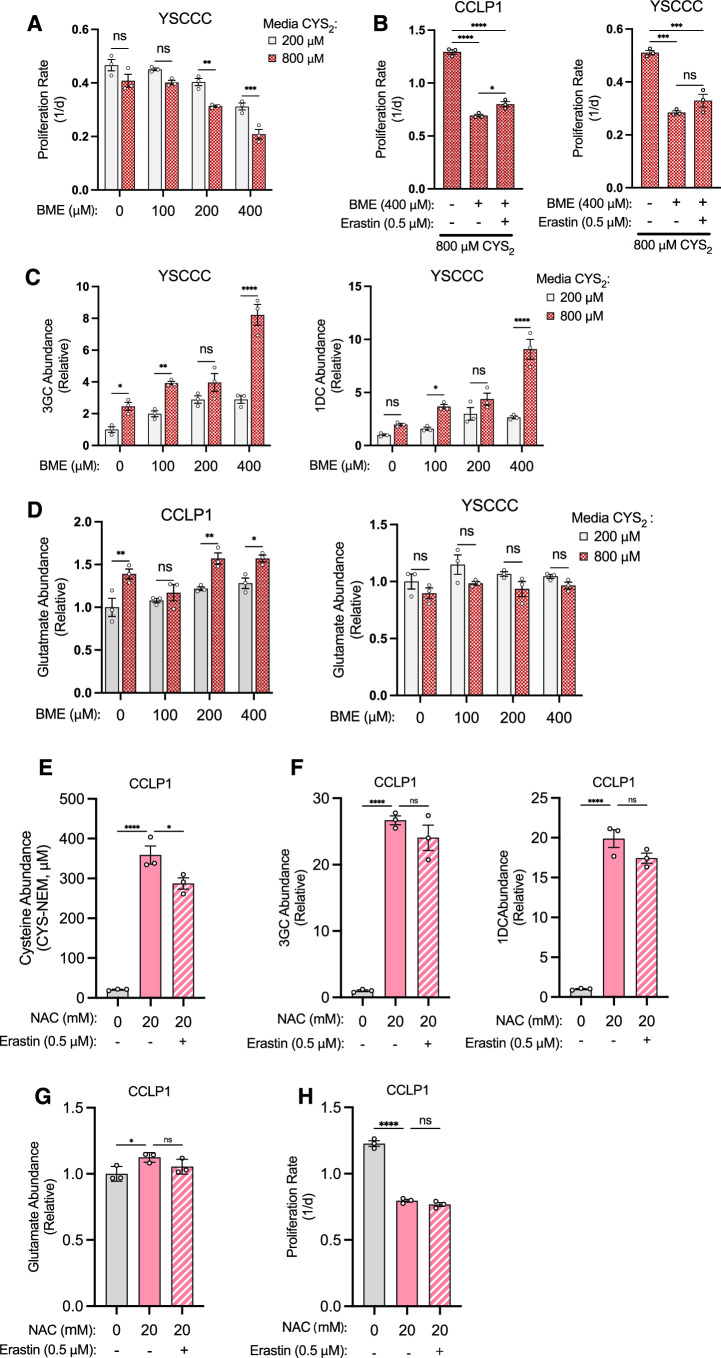
xCT-independent models of excess CYS stress impair cell proliferation and drive cysteine and sugar-CYS conjugate accumulation without glutamate depletion. (**A**) Cell proliferation rates of YSCCC cells treated with different media concentrations of BME or vehicle (ethanol, EtOH), in media containing either 200 or 800 μM CYS_2_. *n* = 3 replicate wells per condition. 0 μM BME *P* = 0.0594, 100 μM BME *P* = 0.1341, 200 μM *P* = 0.0027, 400 μM BME *P* = 0.0007. (**B**) Cell proliferation rates of NRF2^off^ cell lines YSCCC and CCLP1 in media containing 800 μM CYS_2_ treated with vehicle control (EtOH) or 400 μM BME, or with additional vehicle control (DMSO) or 0.5 μM erastin. *n* = 3 replicate wells per condition. CCLP1: 0 μM BME vs 400 μM BME *P* < 0.0001, 0 μM BME vs 400 μM BME + 0.5 μM erastin *P* < 0.0001, 400 μM BME vs 400 μM BME + 0.5 μM erastin *P* = 0.0272. YSCCC: 0 μM BME vs 400 μM BME *P* = 0.0001, 0 μM BME vs 400 μM BME + 0.5 μM erastin *P* = 0.0004, 400 μM BME vs 400 μM BME + 0.5 μM erastin *P* = 0.2316. Relative ion counts of sugar-CYS conjugates (**C**) or glutamate (**D**) as measured by LC–MS metabolomics in YSCCC cells cultured in media containing either 200 or 800 μM CYS_2_ and treated with different media concentrations of BME or vehicle control (EtOH) for 24 h. Relative ion count abundance calculated as relative to the average of 200 μM CYS_2_ treated with vehicle for each metabolite. *n* = 3 replicate wells per condition. 3GC: 0 μM BME *P* = 0.0407, 100 μM BME *P* = 0.0059, 200 μM *P* = 0.1850, 400 μM BME *P* < 0.0001. 1DC: 0 μM BME *P* = 0.4611, 100 μM BME *P* = 0.0184, 200 μM *P* = 0.1644, 400 μM BME *P* < 0.0001. Glutamate, CCLP1: 0 μM BME *P* = 0.0028, 100 μM BME *P* = 0.8112, 200 μM *P* = 0.0065, 400 μM BME *P* = 0.0256. Relative ion counts of cysteine, measured as CYS-NEM, (**E**), sugar-CYS conjugates (**F**), or glutamate (**G**) as measured by LC–MS metabolomics in CCLP1 cells treated with vehicle (H_2_O) or 20 mM N-Acetyl Cysteine (NAC), and with additional vehicle control (DMSO) or 0.5 μM erastin for 24 h and extracted with NEM. *n* = 3 replicate wells per condition. CYS-NEM: 0 vs 20 mM NAC *P* < 0.0001, 20 mM NAC + 20 mM NAC + 0.5 μM erastin *P* = 0.0334. 3GC: 0 vs 20 mM NAC *P* < 0.0001, 20 mM NAC + 20 mM NAC + 0.5 μM erastin *P* = 0.2707. 1DC: 0 vs 20 mM NAC *P* < 0.0001, 20 mM NAC + 20 mM NAC + 0.5 μM erastin *P* = 0.1126. Glutamate: 0 vs 20 mM NAC *P* = 0.0405, 20 mM NAC + 20 mM NAC + 0.5 μM erastin *P* = 0.2288. (**H**) Cell proliferation rates of CCLP1 cells treated with vehicle (H_2_O) or 20 mM N-Acetyl Cysteine (NAC), and with additional vehicle control (DMSO) or 0.5 μM erastin. *n* = 3 replicate wells per condition, 0 vs 20 mM NAC *P* < 0.0001, 20 mM NAC + 20 mM NAC + 0.5 μM erastin *P* = 0.4472. Statistical significance was assessed using two-way ANOVA with Sidak’s correction for multiple comparisons (**A**, **C**-**D**) or by one-way ANOVA with Dunnett’s correction for multiple comparisons (**B**, **E**-**H**). ns = not significant, *P < 0.05, **P < 0.01, ***P < 0.001, ****P < 0.0001. Source data

To uncouple these variables, we sought methods to increase intracellular CYS levels without requiring the glutamate export necessary for xCT-mediated CYS_2_ entry. CYS cannot reliably be added directly to the medium as it is unstable and prone to oxidation. However adding β-mercaptoethanol (BME) to the medium can react with CYS_2_ and enable SLC7A11-independent CYS acquisition (Fig. 3d)^47,48^. We thus cultured CCLP1 and YSCCC cells in 200 μM or 800 μM CYS_2_ with increasing doses of BME to determine whether this co-treatment phenocopies excess CYS_2_ uptake. While both cell lines were resistant to any antiproliferative effects of high CYS_2_ in the absence of BME, the toxic effects of excess CYS were revealed when BME co-treatment enabled dose-dependent CYS acquisition through this alternate route (Fig. 3e and Extended Data Fig. 9a). The antiproliferative effects of BME and CYS_2_ were also not substantially rescued by xCT inhibition, as expected (Extended Data Fig. 9b). LC–MS measurements demonstrated that the proliferation defects from BME-mediated CYS delivery were associated with an increase in sugar–CYS conjugate levels (Fig. 3f and Extended Data Fig. 9c), without causing a depletion in glutamate (Extended Data Fig. 9d). We also tested another method of xCT-independent CYS delivery through treating cells with high dose *N*-acetylcysteine (NAC), which can serve as a CYS prodrug^49^. In CCLP1 cells, NAC treatment also substantially increased levels of CYS and sugar–CYS conjugates, without depleting glutamate (Extended Data Fig. 9e–g). Additionally, NAC impaired cell proliferation, and neither the proliferation defect nor the metabolic changes were prevented by xCT inhibition (Extended Data Fig. 9e–h). These data indicate that excess cysteine acquisition is sufficient to phenocopy the metabolic and functional effects of excess CYS_2_ uptake independent of glutamate depletion. Collectively these results imply that surplus CYS acquisition can drive a state of ‘excess CYS stress’, defined here as a state of CYS overabundance that drives the generation and accumulation of sugar–CYS conjugates and impairs cell proliferation.

**Fig. 4:**
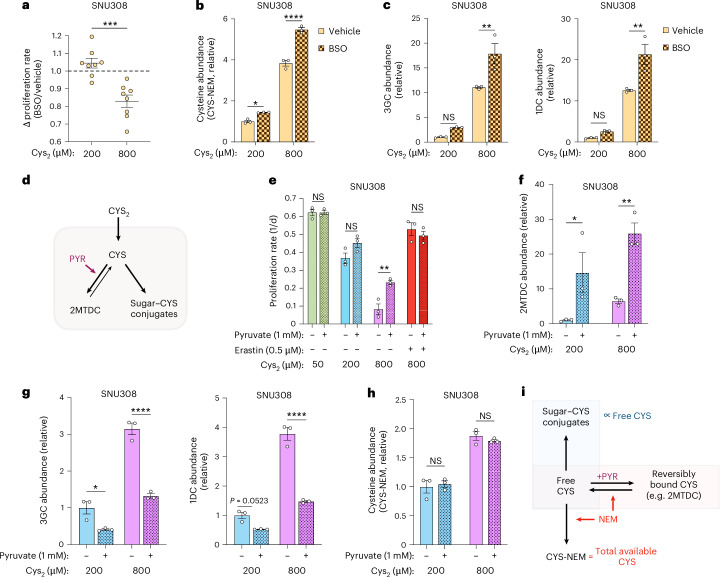
Free cysteine defines the proliferation defects caused by excess cysteine stress. **a**, Change in cell proliferation rate of SNU308 cells cultured in medium containing either 200 or 800 μM CYS_2_ upon co-treatment with 50 μM BSO. Each value represents the average result across technical replicates from *n* = 8 independent experiments, *P* = 0.0003. **b**, Relative abundance of intracellular cysteine, measured as CYS–NEM, from SNU308 cells cultured in medium containing either 200 or 800 μM CYS_2_ treated with either vehicle (DMSO) or 50 μM BSO for 24 h then extracted with NEM and measured by LC–MS metabolomics. Abundances are relative ion counts to the 200 μM CYS_2_ vehicle group. *n* = 3 replicate wells per condition, 200 μM CYS_2_ *P* = 0.0170, 800 μM CYS_2_ *P* < 0.0001. **c**, Relative abundances of intracellular sugar–CYS conjugates as measured by LC–MS metabolomics from SNU308 cells cultured in medium containing either 200 or 800 μM CYS_2_ treated with either vehicle (DMSO) or 50 μM BSO for 24 h. Abundances are relative ion counts to cells cultured in 200 μM CYS_2_ with vehicle for each metabolite. *n* = 3 replicate wells per condition. 3GC: 200 μM CYS_2_ *P* = 0.3876, 800 μM CYS_2_ *P* = 0.003_2_. 1DC: 200 μM CYS_2_ *P* = 0.6299, 800 μM CYS_2_ *P* = 0.00_2_0. **d**, Schematic depicting hypothesized model in which pyruvate treatment can impact the availability of free CYS through sequestration of CYS into 2MTDC. **e**, Cell proliferation rates of SNU308 cells cultured in different medium concentrations of CYS_2_ with or without 1 mM pyruvate, with or without 0.5 μM erastin. *n* = 3 replicate wells per condition. 50 μM CYS_2_ *P* > 0.9999, 200 μM CYS_2_ *P* = 0.1011, 800 μM CYS_2_ *P* = 0.0021, 800 μM CYS_2_ + 0.5 μM erastin CYS_2_ *P* = 0.7829. **f**,**g**, Relative abundances of 2MTDC (**f**) or sugar–CYS conjugates (**g**) as measured by LC–MS metabolomics in SNU308 cells cultured in either 200 or 800 μM CYS_2_ with or without 1 mM pyruvate. *n* = 3 replicate wells per condition. 2MTDC: 200 μM CYS_2_ *P* = 0.0367, 800 μM CYS_2_ *P* = 0.0059. 3GC: 200 μM CYS_2_ *P* = 0.0167, 800 μM CYS_2_ *P* < 0.0001. 1DC: 200 μM CYS_2_ *P* = 0.0523, 800 μM CYS_2_ *P* < 0.0001. **h**, Relative abundance of total intracellular cysteine, measured as CYS–NEM, from SNU308 cells cultured in medium containing either 200 or 800 μM CYS_2_ with or without 1 mM pyruvate for 24 h, then extracted with NEM and measured by LC–MS metabolomics. Abundances are relative ion counts to the 200 μM CYS_2_ vehicle group. *n* = 3 replicate wells per condition. **i**, Schematic depicting a model where sugar–CYS conjugate abundance may proportionately reflect the free CYS pool, while NEM extraction reveals the total available CYS pool, incorporating both the free CYS and the CYS that is sequestered in reversibly bound metabolites (such as 2MTDC). Error bars show s.e.m. Statistical significance was assessed by unpaired two-tailed Student’s *t*-test (**a**) or two-way ANOVA with Sidak’s correction for multiple comparisons (**b**,**c**,**e**–**h**). *P < 0.05, **P < 0.01, ***P < 0.001, ****P < 0.0001. Source data

**Extended Data Fig. 10:**
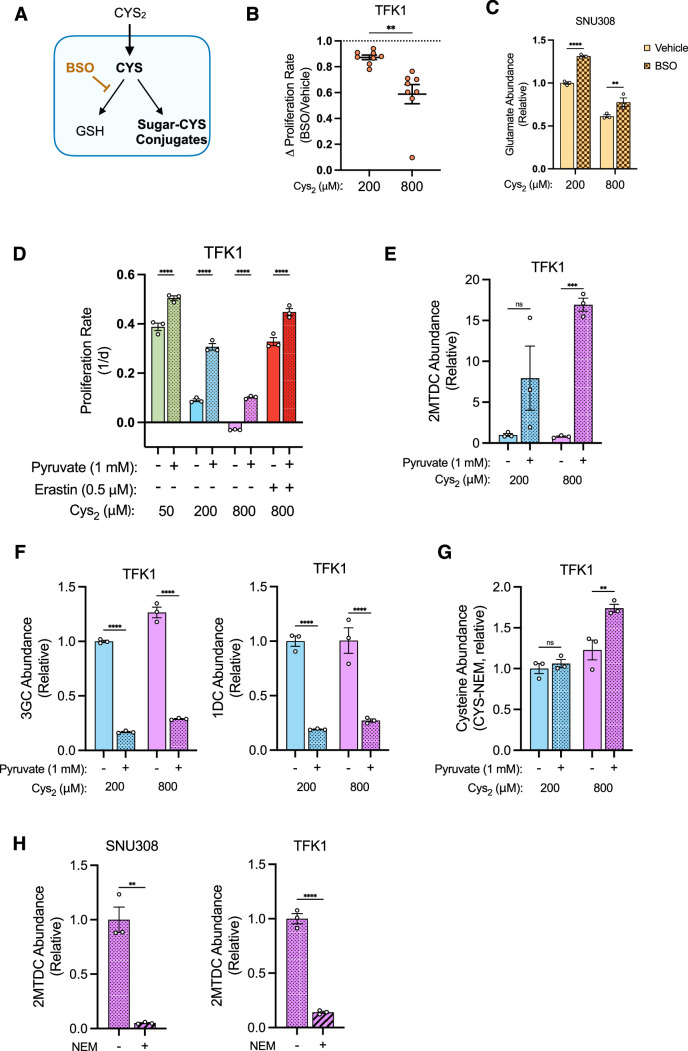
Effects of treatments that alter free intracellular cysteine in cells. (**A**) Schematic depicting hypothesized model of by which BSO treatment can prevent CYS consumption into GSH synthesis, increasing the availability of free CYS and resulting in increased production of sugar-CYS conjugates. (**B**) Change in cell proliferation rate of TFK1 cells cultured in media containing either 200 or 800 μM CYS_2_ upon co-treatment with 50 μM buthionine sulfoximine (BSO). Each value represent the average result across technical replicates from *n* = 8 independent experiments. *P* = 0.0023. (**C**) Relative abundance of glutamate as measured by LC–MS metabolomics in SNU308 cells. Abundances are relative to cells cultured in 200 μM CYS_2_ with vehicle treatment. *n* = 3 replicate wells per condition, 200 μM CYS_2_ *P* < 0.0001, 800 μM CYS_2_ *P* = 0.0064. (**D**) Cell proliferation rate of TFK1 cells cultured in different media concentrations of CYS_2_ with or without 1 mM pyruvate and with or without 0.5 μM erastin. *n* = 3 replicate wells per condition. All comparisons *P* < 0.0001. Relative ion counts of 2MTDC (**E**), sugar-CYS metabolites (**F**), and cysteine (**G**), measured as CYS-NEM, as measured by LC–MS metabolomics in TFK1 cells cultured in either 200 or 800 μM CYS_2_ with or without 1 mM pyruvate for 24 h. *n* = 3 replicate wells per condition. 2MTDC: 200 μM CYS_2_ *P* = 0.0790, 800 μM CYS_2_ *P* = 0.0009. 3GC, 1DC: all comparisons *P* < 0.0001. CYS-NEM: 200 μM CYS_2_ *P* = 0.8254, 800 μM CYS_2_ *P* = 0.0029. (**H**) Relative ion counts of 2MTDC from SNU308 and TFK1 cells cultured in 800 uM CYS_2_ with 1 mM pyruvate for 24 h and extracted with standard extraction solvent or NEM extraction solvent, as measured by LC–MS metabolomics. *n* = 3 replicate wells per condition. SNU308: *P* = 0.0012. TFK1: *P* < 0.0001. Error bars are SEM. Statistical significance was assessed using unpaired two-tailed Student’s t-test (**B**, **H**) or two-way ANOVA with Sidak’s correction for multiple comparisons (**C**-**G**). ns = not significant, **P < 0.01, ***P < 0.001, ****P < 0.0001. Source data

We were next interested in determining how intracellular metabolic engagement of CYS could impact excess CYS stress. First, we considered whether CYS consumption into GSH synthesis might influence the balance of intracellular CYS and proliferation during excess CYS stress (Extended Data Fig. 10a). Of note, impairing GSH synthesis using BSO had minimal effects on the proliferation of SNU308 and TFK1 cells in standard CYS_2_ concentrations, but it intensified the proliferation defects from high CYS_2_ treatment (Fig. 4a and Extended Data Fig. 10b). LC–MS measurements found that BSO treatment further increased intracellular CYS and sugar–CYS conjugates, commensurate with its proliferation-impairing effects (Fig. 4b,c). Notably, slowing glutamate consumption into GSH synthesis also partially restored glutamate levels, further highlighting that glutamate depletion is unlikely to cause the proliferation defects of these cells upon treatment with high CYS_2_ (Extended Data Fig. 10c). Thus, these data indicate that converting CYS into GSH can decrease CYS levels in cells experiencing excess CYS stress, thereby slowing sugar–CYS conjugate production and mitigating the proliferation defects.

Several carbonyl-containing metabolites can reversibly react with CYS, raising the question of how their availability may influence CYS abundance and its functional effects. While most carbonyl generated CYS fates are derived from aldehyde molecules with well-known toxicities, pyruvate is unique among this group as it is cell permeable and non-toxic. We thus investigated whether treatment of cells with pyruvate can influence CYS homeostasis by reversibly trapping it through the formation of 2MTDC (Fig. 4d)^26,28^. Indeed, we found that pyruvate rescued the proliferation defect of NRF2^on^ of SNU308 and TFK1 cells experiencing excess CYS stress (Fig. 4e and Extended Data Fig. 10d). As expected, pyruvate treatment was associated with increased 2MTDC in SNU308 and TFK1 cells, indicating that some of the CYS pool was sequestered into this reversible fate (Fig. 4f and Extended Data Fig. 10e). Consistent with this interpretation, pyruvate treatment also diminished the abundance of the irreversible sugar–CYS conjugates 3GC and 1DC (Fig. 4g and Extended Data Fig. 10f). Of note, quantification of intracellular CYS (which requires NEM capping of thiols during extraction) revealed that the total detectable CYS pool was not depleted by pyruvate treatment (Fig. 4h and Extended Data Fig. 10g). This result is therefore supports a model where pyruvate can sequester CYS into 2MTDC, functionally decreasing the free CYS pool, thereby slowing the production of sugar–CYS conjugates and mitigating the antiproliferative effects of excess CYS stress. While 1DC and 3GC levels serve as surrogate measurements of the free CYS pool, which determines the toxicity of excess CYS stress, CYS–NEM reflects the total chemically available CYS pool as NEM conjugation of CYS shifts the reversibly bound CYS pool towards free CYS and further conjugation (Fig. 4i). Indeed, NEM extraction depleted 2MTDC in both cell lines (Extended Data Fig. 10h). Collectively, these findings reveal that dynamic factors, including CYS consumption processes and reversible biochemical interactions, can shift the balance between free and total CYS, impacting free CYS levels and the magnitude of excess CYS stress.

In this study, we investigated the NRF2-associated metabotype of increased CYS_2_ consumption, which we find occurs without proportionally increased demands on conventional CYS metabolism. Using an untargeted isotope-tracing technique, we identified a set of previously uncharacterized CYS fates with increased abundance in NRF2-activated cells and tumours. While enriched in NRF2-activated cancer cells, these metabolites were also present at lower levels in cells without NRF2 activation, suggesting that the processes that generate them are operative in normal cellular physiology. Herein we identified the biochemical source of several CYS fates, which may serve as biomarkers of excess CYS stress. However, we note that this dataset contains additional features that potentially correspond to other uncharacterized metabolic fates of CYS, highlighting that the CYS metabolome likely remains incomplete. Indeed, this work contributes to a recent slate of metabolite discovery studies which collectively emphasize that the compendium of known metabolites in human cells remains incomplete^1,50–52^.

We also describe the metabolic outcome of excess CYS stress in NRF2-activated cancer cells through its ability to impair cancer cell proliferation, surfacing additional questions about this phenomenon. Toxicity from excess CYS has previously been observed in diverse model systems, with proposed mechanisms including excess reactive oxygen species generation from CYS autoxidation, altered mitochondrial function, disrupted protein folding, increased H_2_S generation, or other effects^49,53–56^. We did not find specific evidence favouring any of these mechanisms, potentially because heterogeneous cell intrinsic metabolic capabilities and environmental contexts likely influence the mechanisms by which excess CYS impairs cell function in different contexts. For instance, we found that free CYS levels can be influenced by enzymatic consumption into GSH synthesis and by chemical reactions that sequester free CYS, highlighting that the many metabolic activities that interface with CYS could modify its effects^21,57,58^. Additional work will be needed to evaluate how regulation of each these cellular processes can collectively impact intracellular free CYS levels and vulnerability to excess CYS stress. Another noteworthy question is whether the functional effects of excess CYS are directly or indirectly tied to the production of the CYS conjugates, which may be addressed, in part, by the establishment of purified chemical standards for these molecules to enable quantitative measurements of their concentrations and metabolic fluxes in physiological contexts and to test their effects independent of excess CYS.

Altered cell metabolism is a hallmark of cancer, which has prompted intensive efforts to identify and exploit the metabolic differences between cancer cells and normal cells for clinical benefit. In the case of constitutive NRF2 activation in cancer, the magnitude and fixity of SLC7A11 expression may thus introduce metabolic vulnerabilities tied to high xCT activity. Indeed, increased xCT activity has been found to promote dependencies on glutaminase, non-essential amino acids, redox homeostasis and glucose metabolism in several cancer models with NRF2-activating mutations^3–6,9,11,44–46^. Because these processes are also critical for many normal cells, establishing a therapeutic window for the disruption of these processes for cancer treatment will require a titration that preserves normal cell viability. Our findings highlight an alternative approach where, rather than attempting to disproportionately starve cancer cells of a universally essential process, we might instead reinforce the metabolic excesses of cancer cells to drive toxicity. Notably, organismal circulating CYS_2_ levels can be affected by biological context, feeding state and nutritional composition^59,60^, and CYS_2_ supplementation has been found to increase tumour xCT activity in a mouse model of NRF2-activated NSCLC^9^, suggesting that interventions to increase circulating CYS_2_ might selectively impact NRF2-driven tumours by driving excess CYS stress. Thus, an important next step will be to determine whether modulation of environmental CYS_2_ levels can be used to promote excess CYS stress in tumours with high xCT activity and whether the state of excess CYS stress can be further leveraged for therapeutic benefit.

## Methods

### Cell culture

Cell lines were acquired from ATCC (H1299, CRL-5803; A549, CCL-185), JCRB Cell Bank (OCUG1, JCRB0191; KKU100, JCRB1568), Takara (HEK293T Lenti-X, 632180), as a gift from S. Saha, Fred Hutch (SNU308, TFK1, SSP25, RBE, YSCCC and CCLP1) or as a gift from M. Linehan, National Cancer Institute (NCI) (UOK262). Cell identities were confirmed using short-tandem repeat profiling and cells were regularly tested to be free of *Mycoplasma* contamination (MycoProbe, R&D Systems). Cells were sustained in Dulbecco’s modified Eagle medium (DMEM) with pyruvate (Corning, MT-50-003-PC) supplemented 3.7 g l^−1^ sodium bicarbonate (Sigma, S6297), 10% heat-inactivated fetal bovine serum (FBS) (Gibco, 26140079 and Cytiva HyClone, SH3039603HI) and 1% penicillin–streptomycin solution (P/S) (Sigma, P4333). Cells were incubated in a humidified incubator at 37 °C and 5% CO_2_.

### Western blotting

Cells were seeded at 0.5–1 × 10^6^ cells per 6-cm plate, depending on cell size. The following day, plates were placed on ice, washed once with 1 ml ice-cold phosphate-buffered saline (PBS) and 100 μl RIPA buffer (Thermo Fisher, J63324-AK) supplemented with Halt protease and phosphatase inhibitor (Thermo Fisher, 78442) was added to the plate and cells were scraped into a microcentrifuge tube using the back of a P1000 pipette tip. Samples were kept on ice for 30 min, then centrifuged at 17,000*g* for 10 min at 4 °C. The supernatant was transferred to a fresh microcentrifuge tube and quantification of protein was performed using a BCA assay (Thermo Fisher, 23225). Samples were denatured using Bolt 4× LDS Sample Buffer (Thermo Fisher, B0007) and Bolt 10× Reducing Agent (Thermo Fisher, B0004) and heated to 95 °C for 5 min, and then gently spun to collect all condensates that formed on the interior of the tube. Samples were then loaded onto a 4–12% SDS–PAGE (Invitrogen, NW04122BOX) and ran at the following voltages and times: 100 V for 10 min, 150 V for 15 min and 165 V for 25 min. After electrophoretic separation, protein was then transferred to a 0.22-mm nitrocellulose blot using iBlot2 transfer stacks (Thermo Fisher, IB23001) and gel transfer device (Thermo Fisher, IB21001) on the P0 setting. The nitrocellulose blot was then Ponceau stained (Sigma, P7170-1L) and cut if probing for more than two proteins with antibodies produced from the same species. Membranes were blocked with 5% bovine serum albumin (Sigma, A4503-100G) dissolved in Tris-buffered saline with 0.1% Tween-20 (TBS-T) and incubated at 4 °C overnight with the following primary antibodies: anti-NRF2 (Cell Signalling, 33649; 1:500 dilution), anti-Vinculin (Sigma-Aldrich, SAB4200729-100UL; 1:10,000 dilution), anti-xCT/SLC7A11 (Cell Signalling, 12691S; 1:1,000 dilution) and anti-NQO1 (Cell Signalling, 62262; 1:5,000 dilution). The following day, membranes were washed three times with TBS-T, and incubated with the secondary antibodies 680RD goat anti-rabbit IgG (Licor, 926-68071; 1:15,000 dilution) and/or 800CW goat anti-mouse IgG (Licor, 926-32210; 1:15,000 dilution) for 1 h. After secondary antibody incubation membranes were washed three times with TBS-T and imaged using a LiCOR Odyssey Near-Infra-red imaging system.

### NRF2^on^ cell line classification

To identify cell lines with chronic NRF2 activation, we obtained publicly available datasets measuring variables relevant to NRF2 status from depmap.org for gene essentiality (21Q4 Chronos scores for *NFE2L2*, *SLC33A1*, *TAPT1* and *SUCO*), gene expression (21Q4 expression for NRF2 target genes *ABCC2*, *ABCC3*, *AKR1B10*, *AKR1C1*, *GCLM*, *GSR*, *ME1*, *NQO1* and *TXNRD1*), NRF2 pathway mutations (*KEAP1*, *NFE2L2* and *CUL3*) and metabolite levels associated with NRF2 activation (NADP^+^, glutathione disulfide (GSSG) and GSH). NRF2 activation score was calculated by adding the standardized expression scores (x_std_) for each of the nine NRF2 target genes using the formula: x_std_ = (x_i_ − x̄)/σ_x_, where x_i_ = expression of gene x in cell line i, x̄ = average expression of gene x across all cell lines and σ_x_ = s.d. of gene x expression across all cell lines. In total, 973 cell lines had measurements of gene essentiality, gene expression and mutations. NRF2^on^ status was defined as cell lines in the top quintile for both NRF2 dependency (Chronos score <−0.3028) and NRF2 activation score (>4.06), yielding 102 NRF2^on^ cell lines and 875 NRF2^off^ cell lines. Cell lines were also classified by annotated cell lineage and NRF2 pathway mutation status, in which a pathway mutation was defined as either a deletion, frameshift, nonsense or splice site mutation in *KEAP1* or *CUL3*, or any missense mutation in *KEAP1*, *CUL3* or *NFE2L2*. Cell lines of each group were then evaluated for correlations between NRF2^on^ status, NRF2 pathway mutations and phenotypes relevant to NRF2 activation, including dependency on *SLC33A1*, *TAPT1* and *SUCO* in all cell lines and metabolite levels for the subset of cell lines with corresponding measurements of NADP^+^ (626/973), GSSG (617/973) or GSH (617/973).

### Medium consumption measurements

To measure the flux of metabolite consumption and excretion, cells were plated at 1–2 × 10^5^ cells per well of six-well dishes, factoring in proliferation rates and cell volumes to capture a similar range of cell-volume hours over the experiment, and incubated in 4 ml DMEM with dialysed FBS in multiple parallel wells and, at each time point, 500 μl of the medium was removed and frozen, and the cells were trypsinized and counted using a Beckman Coulter Counter Multisizer 4. To account for changes in medium metabolite fluxes from increasing cell numbers over time, and to normalize differences in cell size and proliferation rates between cell lines, consumption rates were measured by fitting a linear regression using medium metabolite moles and the area under the growth curve, using total accumulated cell-volume (μl) hours at each time point. Similar protocols were used to measure metabolite consumption upon treatment with 200 μM BSO in DMEM with FBS or in Human Plasma-Like Media (HPLM; Thermo Fisher, A4899101).

#### Media concentrations

After the experiment was completed, 20 μl of each medium sample was extracted with 500 μl 80% HPLC-grade methanol (80:20 methanol:water). Then, 100 μl from that extraction was transferred to a fresh microcentrifuge tube and dried on a Centrivap vacuum concentrator. Samples were reconstituted to 40 μl in 80% methanol containing U-^13^C, U-^15^N labelled canonical amino acid mix (Cambridge Isotopes, MSK-CAA-1), U-^13^C labelled glucose (Cambridge Isotopes, CLM-1396) and U-^13^C labelled lactate (Cambridge Isotopes, CLM-1579) and transferred to vials for measurement by LC–MS. For HPLM experiments, samples were concentrated by 3.6× compared with DMEM samples. Response ratios were determined by dividing the peak area for each metabolite by the peak area for each labelled standard, which was then mapped to a calibration curve for each metabolite to infer concentration. Medium concentrations were then determined by back calculating for each step introducing a dilution. Finally, medium evaporation and minor pipetting errors introduced before resuspension in isotope standard mix were corrected by normalizing to average phenol red peak area.

##### Cell-volume-hours calculations

Cells were trypsinized at *t* = 0 and eight other time points over two experiments, each *n* = 3, ranging from 6 to 120 h, with time points chosen based on the cell volumes and proliferation rates of each cell line, and total cell volume for each well was determined by Coulter Counter. Cell-volume-hours were calculated at time point *t*, using the equation:

$${\int }_{{T}^{0}}^{{T}^{1}}N\left(t\right)=\frac{{N}_{0}}{k\mathrm{ln}\left(2\right)}({2}^{{kT}}-1)$$

Where *N(t)* represents the cell-volume-hours between time points *T*^0^ and *T*^1^, *N*_0_ is the initial total cell-volume (μl), *k* is the proliferation rate (cell-volume doublings per hour) and *T* is the time between time points *T*^0^ and *T*^1^ (hours). Accumulated cell-volume-hours for each well of each time point were determined by adding the calculated cell-volume-hours for that well to the average accumulated cell-volume-hours of the previous time point. Data points were excluded from calculations of medium consumption rates if cells grew to more than 300 μl hours, which was found to slow cell growth rate and is thereby expected to alter metabolic fluxes. Data points were also excluded for the consumption rate calculations for a metabolite and its closely linked metabolite fluxes (for example, glutamate efflux upon cystine depletion) if that metabolite was depleted by 90% or more, which would likely impair uptake rates.

### Medium conditions and treatments

SNU308, KKU100, SSP25, TFK1, OCUG1, RBE, YSCCC, CCLP1, UOK262, A549 and H1299 cells were seeded at 0.1–2 × 10^5^ cells per well in standard medium conditions. The following day, cells were washed in PBS and changed to the assay medium (DMEM) containing regular or dialysed FBS (Sigma, F0392) and various treatments and times, as indicated. For U-^13^C-glucose-tracing experiments, SSP25 cells were washed with PBS and changed into DMEM without glucose, glutamine, pyruvate or sodium bicarbonate (Sigma, D5030), that had been supplemented with standard DMEM concentrations of U-^13^C-glucose (Cambridge Isotopes, CLM-1396), pyruvate (Sigma, P8574), glutamine (Sigma, G5792), sodium bicarbonate (Sigma, S6297), P/S and dialysed FBS for the indicated times. For low-cystine and low-glucose experiments, cell lines were washed twice with PBS and changed into DMEM without glucose, cystine, pyridoxal HCl or riboflavin (US Biological Life Sciences, D9800-02C), which had been supplemented with glucose (Sigma-Aldrich, G7528) at either 25 mM (normal) or 250 μM (low) and L-cystine (Sigma-Aldrich, C6727) at either 200 μM (normal) or 20 μM (low) and standard concentrations of pyridoxal HCl (Sigma-Aldrich, P6155), riboflavin (Sigma, R9504), P/S and dialysed FBS for 6 h before extraction. Other experiments conducted in standard assay medium included metabolite and/protein extractions after 6-h treatments with 5 mM 2-deoxyglucose (Sigma, D8375), 6 mM glutamate (Sigma G8415), 0.5 μM erastin (Cayman Chemical, 17754) or a dose titration of β-mercaptoethanol (Sigma M3148) and 24-h treatments with 50 μM or 200 μM L-buthionine-sulfoximine (BSO) (Sigma, B2515, Selleckchem S9728), a dose titration of CYS_2_ (±0.5 μM erastin), 100 μM KI696 (MedChemExpress, HY-101140) or 20 mM NAC (±0.5 μM erastin) (Sigma, A7250). For pyruvate-free experiments, cell lines were washed twice with PBS and changed into DMEM without pyruvate, phenol red, glucose, L-cystine or L-glutamine (US Biological Life Sciences, D9815), that had been supplemented with 25 mM glucose, 1× GlutaMAX (Gibco, 35050-061), P/S and FBS, with or without pyruvate or L-cystine, treated with or without 0.5 μM erastin. For HPLM experiments, cell lines were washed twice with PBS and changed into HPLM that had been supplemented with P/S and FBS, with or without L-cystine or 0.5 μM erastin.

### Generation of isotope standard mix for CYS related metabolites

A mix of isotopically labelled CYS metabolite standards was generated for benchmarking abundance changes of CYS related metabolites. Immediately after resuspension, 2.5 mM 3,3-D_2_-CYS (Cambridge Isotope Laboratories, DLM-769-0.1) was combined with 1 mM dihydroxyacetone phosphate (DHAP; Cayman Chemical, 34641) to generate labelled 1DC or 1 mM glyceraldehyde-3-phosphate (G3P; Cayman Chemical, 17865) to generate labelled 3GC. These two solutions were then diluted a tenfold volume of NEM extraction solution, 80% methanol with the remaining 20% consisting of 10 mM ammonium formate (Sigma, 70221), pH 7 in HPLC-grade water with 2.5 mM *N*-ethylmaleimide (NEM; Thermo Fisher Scientific, 040526.06) for a final concentration of 2 mM ammonium formate and 0.5 mM NEM) to conjugate residual 3,3-D_2_-CYS and prevent further oxidation. 3,3-D_2_-CYS with G3P solution was then diluted 1:10 in 3,3-D_2_-CYS with DHAP solution. A solution of 75 mM labelled GSH (^13^C_2_, ^15^N (glycine)-labelled glutathione; Cambridge Isotope Laboratories, CNLM-6245-HP-10) was added at 10 µl per 30 ml of previously mixed solution. The final standard mix contained D_2_ labelled 1DC, 3GC, CYS–NEM and CYS_2_ (from spontaneous oxidation), and ^13^C_2_, ^15^N labelled GSH–NEM. This isotopically labelled CYS metabolite standard mix was used at a volume of 40 µl per 1 µl of cell volume when reconstituting dried samples, allowing calculations of response ratios of unlabelled metabolites (from biological extracts) to their labelled versions to correct for matrix effects or loading issues. For metabolites with available purified standards, standard curves were generated using unlabelled cysteine (CYS; Sigma, 30089) and reduced glutathione (GSH; Sigma, G6529), with each solubilized from powder with NEM extraction solution. These compounds were dried and reconstituted with 50 µl of isotopically labelled CYS metabolite standard mix. Then, a six-point tenfold dilution series was prepared for each compound. Response ratios for each compound were generated by dividing the peak area (ion counts) by the corresponding labelled standard and used to generate a standard curve (best fit of linear, power or second-degree polynomial) for each compound to enable calculations of CYS–NEM and GSH–NEM concentrations in biological samples.

### Metabolite extractions

#### Cell lines

At the time of extraction, cells were washed twice with ice-cold blood bank saline on ice and carefully aspirated. Two separate extraction solvent solutions were used depending on the experiment goals: (1) standard extraction solvent, which consists of 80% methanol (80:20 methanol:water) with or without a valine D8 loading standard; or (2) NEM extraction solvent, used for quantitative measurements of thiol metabolites (and their disulfide counterparts), which consists of 80% methanol with the remaining 20% consisting of 10 mM ammonium formate, pH 7 in HPLC-grade water with 2.5 mM NEM, for a final concentration of 2 mM ammonium formate and 0.5 mM NEM. After wash solution was aspirated from cells, 500 μl of either extraction solvent was added to each well and cells were quickly scraped with the back of a P1000 pipette tip and pipetted into a microcentrifuge tube and placed on ice. Samples were centrifuged at 17,000*g* for 5 min at 4 °C, and 350 μl supernatant was transferred to a fresh microcentrifuge tube and dried on a Centrivap vacuum concentrator (Labonco, 10269602). Matching wells for each condition were also counted on the Coulter Counter to determine total average cell volume for each treatment condition. At the time of analysis, cell extracts were resuspended in 80% methanol with or without U-^13^C yeast extract (Cambridge Isotope, ISO1), with or without NEM and/or isotopically labelled CYS metabolite standard mix at a concentration of 28–40 μl solvent per 1 μl cell volume, vortexed at 4 °C for 5 min and centrifuged at 17,000*g* for 5 min at 4 °C.

#### Tumour extracts

LUAD tumour extracts from mice infused with ^13^C_6_-CYS_2_ were previously generated, extracted in NEM extraction solvent as described by Yoon et al., and used without modification^7^. SqCLC samples were described by Stewart et al. and provided as deidentified samples, aside from annotation of the presence of mutations to *KEAP1* or *NFE2L2* (ref. ^41^). Frozen tumour tissues were pulverized with a prechilled Bio-Pulverizer (59012MS, BioSpec). After weighing the tissues, a standard extraction solvent was added to the pulverized tissue for a final concentration of 50 mg tissue per ml extraction, vortexed well, and incubated at −80 °C overnight. Samples were centrifuged at 17,000*g* for 20 min at 4 °C and kept at −80 °C. Before analysis of SqCLC samples, isotopically labelled CYS metabolite standard mix containing NEM was used as a spike-in standard at a 1:4 dilution. In all cases, 20 μl of the metabolite extract supernatant was transferred to an LC–MS vial until analysis. Samples were kept at −80 °C until the time of analysis.

### LC–MS

Metabolite quantitation of resolubilized metabolite extracts was performed using a Q Exactive HF-X Hybrid Quadrupole-Orbitrap Mass Spectrometer equipped with an Ion Max API source and H-ESI II probe, coupled to a Vanquish Flex Binary UHPLC system (Thermo Scientific). Mass calibrations were completed at a minimum of every 5 days in both the positive and negative polarity modes using LTQ Velos ESI Calibration Solution (Pierce). Metabolites were chromatographically separated by injecting a sample volume of 1 μl into a SeQuant ZIC-pHILIC Polymeric column (2.1 × 150 mm 5 mM, EMD Millipore). The flow rate was set to 150 μl min^−1^, autosampler temperature set to 10 °C and column temperature set to 30 °C. Mobile phase A consisted of 20 mM ammonium carbonate and 0.1% (v/v) ammonium hydroxide, and mobile phase B consisted of 100% acetonitrile. The sample was gradient eluted (% B) from the column as follows: 0–20 min: linear gradient from 85% to 20 % B; 20–24 min.: hold at 20% B; 24–24.5 min: linear gradient from 20% to 85% B; 24.5 min to the end: hold at 85% B until equilibrated with ten column volumes. Mobile phase was directed into the ion source with the following parameters: sheath gas of 45, auxiliary gas of 15, sweep gas of 2, spray voltage of 2.9 kV in the negative mode or 3.5 kV in the positive mode, capillary temperature of 300 °C, RF level of 40% and auxiliary gas heater temperature of 325 °C. Mass detection was conducted with a resolution of 240,000 in full-scan mode, with an AGC target of 3,000,000 and maximum injection time of 250 ms. Metabolites were detected over a mass range of 70–1,050 m/z. Quantitation of all metabolites was performed using Tracefinder 4.1 (Thermo Scientific) referencing an in-house metabolite standards library using ≤5 ppm mass error. For inter-tumour comparisons of metabolite abundance, outliers were excluded using the ROUT outlier test Q = 1%. Data from U-^13^C glucose stable isotope-tracing experiments include correction for natural isotope abundance using IsoCor software (v.2.2). For fractional labelling measurements in tumours, only samples with detection of both M + 0 and M + 3 isotopologues were included in calculating labelled fractions.

### RMA tracing for untargeted metabolite identification

#### ^13^C^15^N-cystine tracing

Cells seeded for intracellular metabolite extractions, as described above, and changed to assay medium (DMEM) containing dialysed FBS and a ~1:1 ratio of labelled:unlabelled cystine, accomplished by adding 200 μM ^13^C_6_^15^N_2_ L-cystine (Cambridge Isotopes, CNLM-4244-H-PK) to the medium. Cells were incubated for 24 h, and intracellular metabolites were extracted as described above and analysed by LC–MS.

#### Peak calling

Raw data generated by LC–MS was first processed using Compound Discoverer 3.0 (Thermo Fisher) to make a list of peaks used in downstream analysis. Each peak in this list has information about the exact mass, retention time, and integrated ion count (peak area) observed in each sample. To generate this peak list, data from each polarity was run as separate instances, spectra were selected using a signal-to-noise threshold of 4 and centroids from multiple files were aligned in retention time space using an adaptive curve model, with a maximum retention time shift of 1 min and a mass tolerance of 5 ppm. The resulting aligned centroids were filtered using signal-to-noise threshold of 5. To collapse centroids derived from the same compound (caused by mass defects, isotopologues and adducts), centroids were assigned to a ‘compound’, which we refer to as peaks. Compound assignment was made using settings of a mass tolerance of 5 ppm, an isotope intensity tolerance of 30%, a signal-to-noise threshold of 5 and a minimum peak intensity of 50,000. Finally, these ‘compounds’ were grouped using a mass tolerance of 5 ppm and a retention time tolerance of 0.4 min. Then, peaks missing in some samples were filled in using the fill-gap function with a mass tolerance of 5 ppm and a signal-to-noise threshold of 1.5.

#### Identification and filtering of RMA-tracing candidate peaks

The peak lists generated using Compound Discover were processed using Python scripts to identify the peaks fulfilling the criteria under the RMA-tracing scheme. A detailed description of the parameters used can be found on GitHub at https://github.com/krdav/RMA_tracing/blob/main/peak-pair-analysis_bile-duct-cells_cys-tracing.ipynb

First, the peak list was filtered such that all peaks had at least one sample with an ion count over 25,000, at least one sample receiving labelled cystine with an ion count over 15,000 and at least one sample with an ion count fourfold higher than that of any blanks. Peaks with an exact mass of less than 120 Da (one Dalton below cysteine) were also discarded. A number of these peaks were found to be derived from small mass defects that were not collapsed by Compound Discoverer, giving rise to multiple peaks from the same molecule. These situations were identified when two criteria were both fulfilled: (1) an exact mass difference of ≤50 ppm; and (2) a retention time difference of ≤0.1 min or a retention time difference of ≤0.2 min and a Pearson correlation coefficient between the two peak areas across samples of ≥0.9. Upon identification, peaks were merged by taking the sum of the peak areas and keeping the exact mass and retention time from the peak with the largest sum of peak areas. After this filtering, RMA-tracing candidate peaks were found by searching for their cysteine labelled m + 4 isotopologue peaks using a maximum difference between theoretical and observed exact mass of 10 ppm, a maximum retention time difference of 0.25 min and a labelling fraction range of 0.2–0.28 or 0.34–0.42 observed in at least one sample. These labelling ratio ranges were determined using the empirically measured M + 4/M + 0 ratios of known cysteine fates (for example, 2SC, GSH and lactoylglutathione), with the higher range reflecting the expected and measured labelling fraction of molecules incorporating two cysteines (for example, GSSG). These labelling fractions were lower than the expected ~0.5 labelling ratio, possibly reflecting labelling dilution from residual unlabelled cysteine in these cells or contributions of biosynthetic pathways to the cysteine pool from unlabelled sources. The RMA-tracing candidates were compiled as a list of peak pairs with the unlabelled and m + 4 isotopologue peaks and the peak area for each sample. A number of these peak pairs were found to be adducts or isotopologues of a parent peak pair that was not filtered out in previous steps and thus giving rise to multiple peaks from the same molecule. For adducts, potential adduct masses for each peak pair were calculated using a list of known common adducts. Peak pairs with matching exact mass and retention time were identified and flagged as potential adducts using an exact mass difference of less than or equal to 200 ppm and a retention time difference of ≤0.8 min. For isotopologues, potential isotopologue masses for each peak pair were calculated using a list of the most abundant isotope combinations. Peak pairs with matching exact mass and retention time were identified and flagged as potential isotopologues using an exact mass difference of ≤200 ppm, a retention time difference of ≤0.4 min, a Pearson correlation coefficient between the peak area of the predicted isotopologue and its parent across samples ≥0.7 and a requirement that the peak area of the predicted isotopologue be less than that of its parent.

Python scripts for identification and filtering of RMA-tracing candidate peaks can be found in the associated GitHub repository at https://github.com/krdav/RMA_tracing

#### Stringent filtering of candidate peaks

To generate the final peak list, peak pairs previously flagged as potentially arising from natural isotopes, adducts, known fragments and peak duplicates were removed, and the peak list was subjected to another round of stringent filtering. Stringent filtering was informed by the characteristics of known cysteine fates within the peak list and removed peak pairs with larger deviations in retention times and ppm error from expected masses. As none of the known cysteine fates in the dataset had labelled/unlabelled pairs with a ∆ppm of >0.66 or a ∆RT > 0.04 min, a stringent filter was set to exclude peak pairs with a ∆ppm of greater than or equal to 1.0 or with a retention time difference of greater than or equal to 0.06 min. Finally, for peaks detected in both positive and negative modes the peak with the lower ion count was discarded, yielding the peak final list (Supplementary Table 1).

### Generation of CYS fates by combining CYS with purified metabolites

Chemical standards of glucose fates (sugar standards) were solubilized in PBS at 4 mM; 3-phosphoglycerate (Cayman Chemical, 20123), ribose-5-phosphate (R5P) (Sigma, R7750), ribulose-5-phosphate (Cayman Chemical, 21423), glucose-6-phosphate (Cayman Chemical, 20376), glucose-1-phosphate (Cayman, 30566), xylulose-5-phosphate (Sigma, 15732), 2-phosphoglycerate (Sigma, 79470), glyceraldehyde-3-phosphate (G3P) (Cayman Chemical, 17865), dihydroxyacetone phosphate (DHAP) (Cayman Chemical, 34641), phosphoenolpyruvate (PEP) (Cayman Chemical, 19192-250) or 2 mM methylglyoxal (Sigma, M0252), DL-lactaldehyde (Sigma, 49426), hydroxyacetone (Sigma, 138185), sodium lactate (Sigma, 71719) and sodium pyruvate (Sigma, P8574). L-cysteine (Sigma-Aldrich, 30089) was prepared fresh by dissolving in a half volume of 1 M HCl, neutralized with a half volume of 1 M NaOH, and diluted to a 10 mM stock solution in PBS. Sugar standards were then either extracted immediately or combined with L-cysteine in a PCR tube at equal volumes to achieve a final reaction concentration of 5 mM L-cysteine with 1–2 mM sugar standards in duplicate. Reactions were then incubated overnight in a PCR machine at 37 °C and extracted the following day and prepared for LC–MS analysis. To measure the stoichiometry of reactions between cysteine and sugar phosphate standards, 10 mM stocks of DHAP, G3P, and R5P were diluted to 1 mM stock solutions in PBS and placed on ice. A neutralized stock of freshly prepared L-cysteine was generated as above, diluted to 10 mM, 5 mM, 2 mM, 1 mM, 0.5 mM 0.25 mM or 0 mM in PBS, and dispensed into microcentrifuge tubes. Equal volumes of 1 mM stocks of DHAP, G3P or R5P were then added to each tube, to achieve final concentrations of 5 mM, 2.5 mM, 1 mM, 0.5 mM, 0.125 mM and 0 mM L-cysteine and 500 μM DHAP, R5P and G3P. They were then pipette mixed ten times, spun gently, incubated at 37 °C for 1 h and returned to ice. To measure the production of CYS fates with other carbonyls, a reaction solution containing 5 mM L-cysteine and 0.5 mM solution of formaldehyde (Sigma, F1635), acetaldehyde (Sigma, 402788), formic acid (Sigma, 5.33002), acetone (Sigma, 270725-2L) or pyridoxal (Sigma, P6155-5G) in PBS was incubated for 1 h. After incubation, reactions were then dried on a Centrivap concentrator and resuspended at the reaction volume in 80% HPLC-grade methanol (80:20 methanol:water) when ready for analysis, transferred to an LC–MS vial, and submitted for LC–MS measurements.

### Lentiviral production and infection

The following plasmids were obtained from Addgene: pMDLg/pRRE (12251, a gift from D. Trono), pMD2.G (12259, a gift from D. Trono), pRSV-Rev (12253, a gift from D. Trono), pDONR223_NFE2L2_p.G31R (81520, a gift from J. Boehm, W. Hahn and D. Root), pInducer20 (44012, gift from S. Elledge), lentiMPHv2 (89308, gift from F. Zhang) and LentiSAMv2 (75112, gift from F. Zhang). NFE2L2_p.G31R was cloned from pDONR223 to pInducer20 using LR Clonase II (Fisher, 11791100). *SLC7A11* was cloned via CRISPR Activation. Guide RNAs (gRNAs) were selected for the promoter region of human *SLC7A11* using CRISPick software (https://portals.broadinstitute.org/gppx/crispick/public) and the *SLC7A11* gRNA oligonucleotide (5’–3’: AAAGAGCTGAGTAATGCTGG) was modified according to *BsmbI* restriction sites on the customizable lentiSAMv2 plasmid and purchased from Integrated DNA Technologies. LentiSAMv2 was digested to create *BsmbI* overhangs, and the *SLC7A11* gRNAs were annealed and cloned into the digested vector, and verified using whole-plasmid sequencing (Plasmidsaurus). Lentivirus was generated by transfection of HEK293T cells with expression construct plasmid DNA along with pMDLg/pRRE, pRSV-Rev and pMD2.G packaging plasmids with FuGENE transfection reagent (Fisher, PRE2693) in DMEM (Fisher, MT10017CV) without FBS or P/S. The supernatant containing lentiviral particles was filtered through 0.45-µM membrane (Fisher, 9720514) and was supplemented with 8 µg µl^−1^ Polybrene (Sigma, TR-1003-G) before infection. Cells were cultured to ~20–50% confluency in six-well dishes and centrifuged with lentivirus-containing medium (900*g*, 90 min, 30 °C). *SLC7A11* overexpressing cells received both lentiMPHv2 and lentiSAMv2-SLC7A11 viruses. After 24 h, cells were replenished with fresh medium and after 48 h, cells were selected with 1 or 10 μg ml^−1^ blasticidin (Fisher, R21001), 150 µg µl^−1^ hygromycin (Sigma, H7772), 0.8 mg ml^−1^ G418 (Sigma, A1720) and maintained in selection medium until all uninfected control cells had died.

### Proliferation assays

Exponentially growing cells were seeded overnight in standard DMEM onto six-well dishes (Corning, 3516) with an initial seeding density of 0.2–2 × 10^5^ cells per well, based on cell size and proliferation rates. After overnight incubation, replicate wells were trypsinized and counted for a starting cell count at the time of treatment. Remaining cells were washed twice in PBS and 4 ml of treatment medium was added. For all proliferation experiments except NAC treatments, medium was refreshed on day 2. All final counts occurred on day 4. Additional conditions include a 24-h pretreatment (before day 0 counts) with 100 μM KI696 (MedChemExpress, HY-101140) or co-treatment with 0.5 μM erastin (Cayman Chemical, 17754). Proliferation rate was determined by the following equation: proliferation rate (doublings per day, 1/d) = (log_2_(final cell count/initial cell count))/total days.

### Bioenergetic measurements

Oxygen consumption and extracellular acidification measurements were conducted using an Agilent Seahorse Xfp Analyzer. SNU308, TFK1 or CCLP1 cell lines were trypsinized and seeded overnight at 0.1–1 × 10^6^ cells, depending on cell size, in 100 μl of medium in XF96 cell culture microplates (Agilent, 101085-004). The following day, cells were washed once with PBS and 200 μl of DMEM supplemented with P/S and FBS was added, with or without 0.5 μM erastin and 200 μM or 800 μM CYS_2_. Additionally, the sensor cartridge was incubated overnight in H_2_O. The morning after, cells were washed twice with PBS and 180 μl of DMEM without bicarbonate and without FBS was added. CYS_2_ and erastin treatment was maintained. At this time, the sensor cartridge was switched to be incubated in calibrant solution (Agilent 100840-000). The sensor cartridge was loaded with an injection solution yielding a final concentration of 0.5 μM rotenone (rot) (Sigma, R8875) and 0.5 μM antimycin A (AA) (Sigma, A8674). Following the assay, the medium was removed and 10 μl of RIPA buffer was added directly to each well and a BCA assay was conducted to quantify protein in each well. All measurements were normalized to μg of protein. Basal oxygen consumption and extracellular acidification rates were determined by the pre-injector measurements. Non-mito oxygen consumption was calculated to be the oxygen consumption after rot/AA treatment.

### Statistics

Identified cysteine fate abundances were normalized to *z* scores across bile duct cancer cell lines and principal-component analysis was conducted in GraphPad Prism v.10. Statistical tests used across experimental groups are annotated in each figure legend and were conducted in GraphPad Prism v.10. Sample sizes were not predetermined but were based on observed variance in standard measurements (LC–MS experiments, proliferation assays and Seahorse measurements) or by including all available samples (Depmap datasets and tumour samples). Data distribution was assumed to be normal but this was not formally tested. When possible, samples groups were randomized in the order of analysis to distribute systemic errors. Data collection and analysis were not performed blind to the conditions of the experiments. All non-tumour experiments were repeated at least once with qualitatively similar results. For murine and human tumour LC–MS metabolite abundance data, a ROUT outlier test (Q = 1%) was performed before the data were normalized. All measurements shown are from distinct samples, with data points representing technical replicates from parallel conditions on the same experiment, unless stated otherwise.

### Reporting summary

Further information on research design is available in the Nature Portfolio Reporting Summary linked to this article.

## Supplementary information

Supplementary Information Supplementary Fig. 1. Chemical synthesis procedure for 1DC/3GC and LC–MS results.

Reporting Summary

Supplementary Table 1 Summary of RMA-tracing hits, their confirmed or inferred chemical identities and abundance across cell lines and conditions.

## Source data

Source Data Fig. 1 Source data and statistical test results for Fig. 1.

Source Data Fig. 1 Unprocessed western blots.

Source Data Fig. 2 Source data and statistical test results for Fig. 2.

Source Data Fig. 3 Source data and statistical test results for Fig. 3.

Source Data Fig. 4 Source data and statistical test results for Fig. 4.

Source Data Extended Data Fig. 1 Source data and statistical test results for Extended Data Fig. 1.

Source Data Extended Data Fig. 2 Source data and statistical test results for Extended Data Fig. 2.

Source Data Extended Data Fig. 3 Source data and statistical test results for Extended Data Fig. 3.

Source Data Extended Data Fig. 4 Source data and statistical test results for Extended Data Fig. 4.

Source Data Extended Data Fig. 5 Source data and statistical test results for Extended Data Fig. 5.

Source Data Extended Data Fig. 6 Source data and statistical test results for Extended Data Fig. 6.

Source Data Extended Data Fig. 7 Source data and statistical test results for Extended Data Fig. 7.

Source Data Extended Data Fig. 8 Source data and statistical test results for Extended Data Fig. 8.

Source Data Extended Data Fig. 9 Source data and statistical test results for Extended Data Fig. 9.

Source Data Extended Data Fig. 10 Source data and statistical test results for Extended Data Fig. 10.

## Data Availability

All data supporting the findings of this study are available within the paper and its source data files. RMA-tracing peak lists and relevant secondary results are provided in Supplementary Table 1. Unprocessed western blot data, data used to generate all figures and statistical test results are included in the source data files. Source data are provided with this paper.

## Code availability

The code used to perform RMA tracing is provided on GitHub (https://github.com/krdav/RMA_tracing).
