# Supplementary material for: Excess cysteine drives conjugate formation and impairs proliferation of NRF2-activated cancer cells
Source: Nat Metab. 2026 Apr 7;8(4):840–54. doi: 10.1038/s42255-026-01499-8 (PMC13121033; doi:10.1038/s42255-026-01499-8)
Supplement: Supplementary file 1 — Supplementary Fig. 1. Chemical synthesis procedure for 1DC/3GC and LC–MS results. [file 42255_2026_1499_MOESM1_ESM.pdf]

nature metabolism

Supplementary information https://doi.org/10.1038/s42255-026-01499-8

Excess cysteine drives conjugate formation and impairs proliferation of NRF2-activated cancer cells

In the format provided by the authors and unedited

Supplementary Information

This file describes chemical synthesis approaches to generate 1DC and 3GC from orthogonal chemical precursors. Reagents were purchased from the commercial vendors later specified and used without further purification, unless otherwise detailed. ACS grade solvents were used for flash column chromatography and reaction when indicated: EtOAc (Fisher, ACS grade, Part. No. E145-20), hexane (Fisher, ACS grade, Part No. H292-20).

Flash column chromatography was performed on a Teledyne CombiFlash Nextgen 300+ using Silicycle SiliaSep cartridges. Thin layer chromatography (TLC) was performed using Merck silica gel 60 F254 glass plates (Part No. 1003900001) and visualization was accomplished with UV light (254 nm) and/or staining with basic $KMnO_4$ solution (4 g of $KMnO_4$, 10 g K_2CO_3, 1 g NaOH in 200 ml of distilled water).

HPLC purification was performed on a Waters Autopure system (XBridge C18 5 μm 4.6 x 150 mm) or Waters Acquity system (Acquity UPLC BEH C18 1.7 μm 2.1 x 100 mm) equipped UV-vis, MS, and ELS detectors using MeCN+0.1% formic acid and H_2O+0.1% formic acid gradients.

^{1}H NMR spectra were recorded at the NMR core facility at Albert Einstein College of Medicine at room temperature on a Bruker Avance III HD 300 in $CDCl_3$ (Fisher, >99.8% d, Part No. 166251000). Chemical shifts (δ) for ^{1}H NMR spectra are given in parts per million (ppm) relative to tetramethylsilane (TMS) using the residual solvent signals as reference ($CDCl_3$: δH = 7.26 ppm). NMR-signals multiplicities that can be analyzed as first order multiplets are reported using the following abbreviations (or combination thereof): s = singlet, d = doublet, t = triplet, q = quartet, p = quintet, h =sextet; hept = heptet; m = multiplet, br = broad. All spectra were processed using MestReNova 14 using standard phase and baseline correction automations.

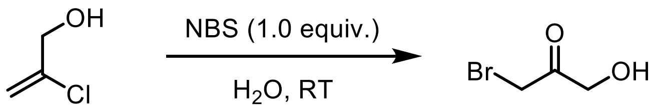

1-Bromo-3-hydroxypropanone. The reaction was adapted from a literature report by Drueckhammer and co-workers (*Tetrahedron Letters* **1993**, *34*, 1733-1736). In a 500-mL round-bottom flask equipped with a PTFE-coated stirring bar, *N*-bromosuccinimide (9.62 g, 54.0 mmol, 1.0 equiv.) (TCI, B0656) was suspended in deionized water (270 mL), then 2-chloroprop-2-en-1-ol (4.30 mL, 54.0 mmol, 1.0 equiv.) (Aladdin Scientific, C171127) was added dropwise over one minute under vigorous stirring. The reaction was stirred at room temperature for 30 minutes, the CH_2Cl_2 (200 mL) (Fisher, D3720) was added, the biphasic system was transferred to a separating funnel, and the layers were separated.

The aqueous layer was extracted once with CH_2Cl_2 (100 mL) (Fisher, D3720), then the combined organic layers were dried over anhydrous $MgSO_4$ (Sigma, M7506), filtered, and the solvent was removed by rotary evaporation (700 mbar at 25 °C, then 10 mbar at 25 °C). The crude product was purified by flash column chromatography on silica (40 g SiO_2, 30 to 35% EtOAc in hexane), affording the product (1.33 g, 16%) as a faint yellow oil which solidifies upon storage in the freezer. **^{1}H NMR** ($CDCl_3$, 300 MHz) δ ppm: 4.55 (s, 2H), 3.95 (s, 2H). ***R_f*** (33% EtOAc in hexane): 0.25. The experimental data agrees with the literature reports (*Tetrahedron Letters* **1993**, *34*, 1733-1736).

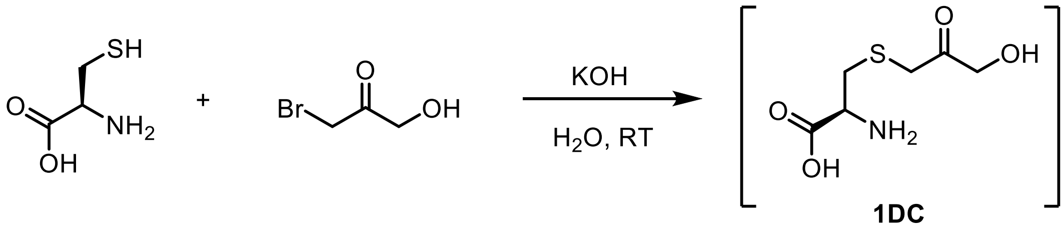

1DC

Chemical synthesis of 1DC. In a 4-mL scintillation vial equipped with a PTFE-coated stirring bar and screw-in septum cap, 1-bromo-3-hydroxypropanone (17.7 mg,116 μmol, 1.0 equiv.) and cysteine (28.0 mg, 231 μmol, 2.0 equiv) (Thermo, J63745.22) were dissolved in deionized water (600 μL), then aqueous KOH (2 M, 100 μL) (Sigma, 221473) was added, and the reaction was thoroughly degassed by freeze-pump-thaw (3x) and then stirred at room temperature for 4 hours. The reaction was transferred to a test tube with MeCN/H_2O (1/1, 1 mL) and lyophilized. The reaction was reconstituted in D_2O (Sigma, 151882) for analysis.

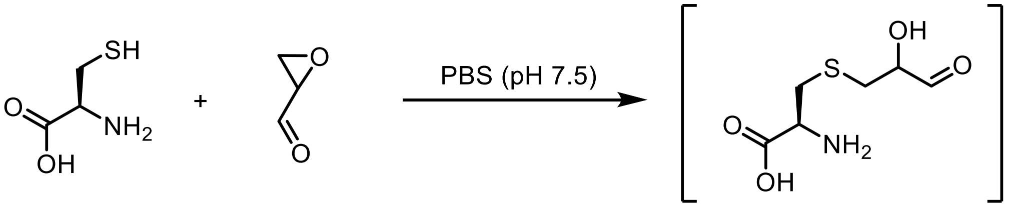

3GC

Chemical synthesis of 3GC. In a 4-mL scintillation vial equipped with a PTFE-coated stirring bar and screw-in septum cap, oxirane-2-carbaldehyde (17.7 mg, 0.13 mmol, 1.0 equiv.) (Enamine, EN300-103531) and cysteine (32.0 mg, 0.27 mmol, 2.0 equiv) (Thermo, J63745.22) were dissolved PBS pH 7.5 (600 μL), and the reaction was thoroughly degassed by freeze-pump-thaw (3x) and then stirred at room temperature for 4 hours. The reaction was transferred to a test tube with MeCN/H_2O (1/1, 1 mL) and lyophilized. The reaction was reconstituted in D_2O (Sigma, 151882) for analysis.

LC-MS of synthesized 1DC and 3GC. 1DC and 3GC solutions were dried by Centrivap vacuum concentrator and resuspended in 80% methanol. Samples were loaded by standard LC-MS protocol (see methods) and relevant m/z peaks were compared to LC-

MS data from cell extracts of A549 cells to compare synthesized peaks to those from a NRF2^{on} cell line and account for retention time drift (approximately 0.5 minutes earlier across all analytes, compared to earlier runs). The resulting chromatography for synthesized 1DC/3GC shows dominant peaks aligning with those from A549 cells (Supplementary Information Figure 1)

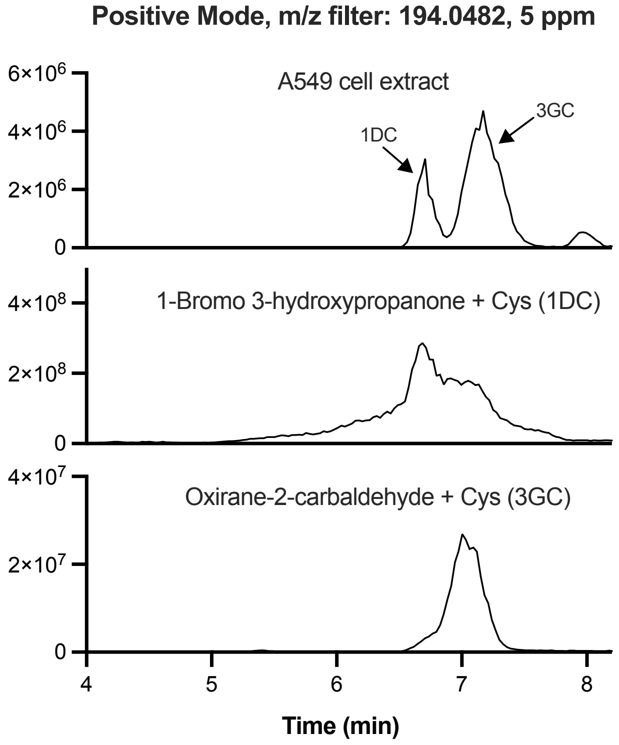

Supplementary Information Figure 1: LC-MS chromatography for isobaric peaks from samples of A549 cell extract compared to the product of cell-free reactions described above, from combining CYS with either 1-Bromo-3-hydroxypropanone (generating 1DC) or oxirane-2-carbaldehyde (generating 3GC).
