# Supplementary material for: Excess cysteine drives conjugate formation and impairs proliferation of NRF2-activated cancer cells
Source: Nat Metab. 2026 Apr 7;8(4):840–54. doi: 10.1038/s42255-026-01499-8 (PMC13121033; doi:10.1038/s42255-026-01499-8)
Supplement: Supplementary file 2 — Reporting Summary [file 42255_2026_1499_MOESM2_ESM.pdf]

nature portfolio

Corresponding author(s): Lucas Sullivan

Last updated by author(s): Feb 12, 2026

Reporting Summary

Nature Portfolio wishes to improve the reproducibility of the work that we publish. This form provides structure for consistency and transparency in reporting. For further information on Nature Portfolio policies, see our Editorial Policies and the Editorial Policy Checklist.

Statistics

For all statistical analyses, confirm that the following items are present in the figure legend, table legend, main text, or Methods section.

n/a	Confirmed	
☐	☒	The exact sample size (*n*) for each experimental group/condition, given as a discrete number and unit of measurement
☐	☒	A statement on whether measurements were taken from distinct samples or whether the same sample was measured repeatedly
☐	☒	The statistical test(s) used AND whether they are one- or two-sided<br>*Only common tests should be described solely by name; describe more complex techniques in the Methods section.*
☐	☒	A description of all covariates tested
☐	☒	A description of any assumptions or corrections, such as tests of normality and adjustment for multiple comparisons
☐	☒	A full description of the statistical parameters including central tendency (e.g. means) or other basic estimates (e.g. regression coefficient) AND variation (e.g. standard deviation) or associated estimates of uncertainty (e.g. confidence intervals)
☐	☒	For null hypothesis testing, the test statistic (e.g. *F*, *t*, *r*) with confidence intervals, effect sizes, degrees of freedom and *P* value noted<br>*Give P values as exact values whenever suitable.*
☒	☐	For Bayesian analysis, information on the choice of priors and Markov chain Monte Carlo settings
☒	☐	For hierarchical and complex designs, identification of the appropriate level for tests and full reporting of outcomes
☒	☐	Estimates of effect sizes (e.g. Cohen's *d*, Pearson's *r*), indicating how they were calculated

Our web collection on statistics for biologists contains articles on many of the points above.

Software and code

Policy information about availability of computer code

Data collection	*Provide a description of all commercial, open source and custom code used to collect the data in this study, specifying the version used OR state that no software was used.*
Data analysis	Software: Compound Discoverer 3.0; LCMS peak list generation and quantification , Tracefinder 4.1; LCMS peak quantification. Natural Abundance correction; IsoCore v.2.2. Code: RMA tracing algorithm

For manuscripts utilizing custom algorithms or software that are central to the research but not yet described in published literature, software must be made available to editors and reviewers. We strongly encourage code deposition in a community repository (e.g. GitHub). See the Nature Portfolio guidelines for submitting code & software for further information.

Data

Policy information about availability of data

All manuscripts must include a data availability statement. This statement should provide the following information, where applicable:

- Accession codes, unique identifiers, or web links for publicly available datasets
- A description of any restrictions on data availability
- For clinical datasets or third party data, please ensure that the statement adheres to our policy

All data supporting the findings of this study are available within the paper and its Supplementary Information. NRF2on cell line classification data are provided in

Supplementary Table 1. RMA tracing peak lists and relevant secondary results are provided in Supplementary Table 2. Unprocessed western blot data are provided as Source Data Files. Source data for all figures and associated statistical tests are in Source Data Files.

Research involving human participants, their data, or biological material

Policy information about studies with human participants or human data. See also policy information about sex, gender (identity/presentation), and sexual orientation and race, ethnicity and racism.

Reporting on sex and gender	Cell Line biological sex information is provided below.
Reporting on race, ethnicity, or other socially relevant groupings	N/A
Population characteristics	N/A
Recruitment	N/A
Ethics oversight	N/A

Note that full information on the approval of the study protocol must also be provided in the manuscript.

Field-specific reporting

Please select the one below that is the best fit for your research. If you are not sure, read the appropriate sections before making your selection.

☒ Life sciences ☐ Behavioural & social sciences ☐ Ecological, evolutionary & environmental sciences

For a reference copy of the document with all sections, see nature.com/documents/nr-reporting-summary-flat.pdf

Life sciences study design

All studies must disclose on these points even when the disclosure is negative.

Sample size	Sample sizes were chosen based on effect sizes from preliminary data and standard approaches in the field.
Data exclusions	Limited cases of data exclusion are reported in the methods section. Two situations arise: For LUAD fractional labeling samples, 5 fractional labeling samples (Figure 3B, 1DC) were excluded when an isotopologue ratio could not be determined, due to either M+0 or M+3 isotopologues not being detected. For metabolite tumor abundance data outliers were excluded using a ROUT outlier test (Q = 1%), resulting in 3 samples being excluded for Figure 3C (1DC and 3GC) and 9 samples being excluded from Figure 3E (1DC and 3GC). All instances of data exclusion are described in the methods and are clearly indicated in the relevant source data files.
Replication	All experiments were qualitatively repeated several times, or (as in the case of the RMA tracing dataset) primary results were verified as controls in subsequent experiments. For mouse and human tumor samples, tissue samples were not available to repeat the experiment in independent sample sets.
Randomization	Sample groups were randomized in the order of analysis (e.g. proliferation assays, LCMS) to distribute systemic errors.
Blinding	N/A

Reporting for specific materials, systems and methods

We require information from authors about some types of materials, experimental systems and methods used in many studies. Here, indicate whether each material, system or method listed is relevant to your study. If you are not sure if a list item applies to your research, read the appropriate section before selecting a response.

Materials & experimental systems

n/a	Involved in the study	
☐	☒	Antibodies
☐	☒	Eukaryotic cell lines
☒	☐	Palaeontology and archaeology
☒	☐	Animals and other organisms
☒	☐	Clinical data
☒	☐	Dual use research of concern
☒	☐	Plants

Methods

n/a	Involved in the study	
☒	☐	ChIP-seq
☒	☐	Flow cytometry
☒	☐	MRI-based neuroimaging

Antibodies

Antibodies used	Antibody sources, product numbers, and dilutions are provided in the method section.
Validation	Antibodies were verified by orthogonal expression analysis (i.e. correlation with RNA seq measurements NRF2 target genes, SLC7A11, NQO1), overexpression constructs (NRF2, SLC7A11), and chemical activators (NRF2, SLC7A11).

Eukaryotic cell lines

Policy information about cell lines and Sex and Gender in Research

Cell line source(s)	Sources and biological sex:<br>ATCC - H1299, CRL-5803, male<br>ATCC - A549, CCL-185, male<br>JCRB Cell Bank - OCUG1, JCRB0191, male<br>JCRB Cell Bank - KKU100, JCRB1568, female<br>Takara - HEK293T Lenti-X, 632180, female<br>Gift from Dr. Supriya Saha, Fred Hutch - SNU308; unspecified sex, TFK1; male, SSP25; female, RBE; female, YSCCC; female, CCLP1;female.<br>Gift from Dr. Marston Linehan, NCI - UOK262; female
Authentication	Commonly used cell lines were verified by STR profiling (H1299, A549, HEK293T). Secondary features were also used to support the identity of several cell lines (FH deficiency and high GSF levels (UOK262), IDH mutation associated with 2HG accumulation (RBE), and RNAseq correlations with NRF2 activation.
Mycoplasma contamination	All cell lines were regularly tested for mycoplasma contamination and were found to be negative.
Commonly misidentified lines<br>(See ICLAC register)	None of the cell lines used here are commonly misidentified.

Plants

Seed stocks	N/A
Novel plant genotypes	N/A
Authentication	N/A
