# Supplementary material for: Excess cysteine drives conjugate formation and impairs proliferation of NRF2-activated cancer cells
Source: Nat Metab. 2026 Apr 7;8(4):840–54. doi: 10.1038/s42255-026-01499-8 (PMC13121033; doi:10.1038/s42255-026-01499-8)
Supplement: Supplementary file 8 — Source data and statistical test results for Fig. 4. [file 42255_2026_1499_MOESM8_ESM.pdf]

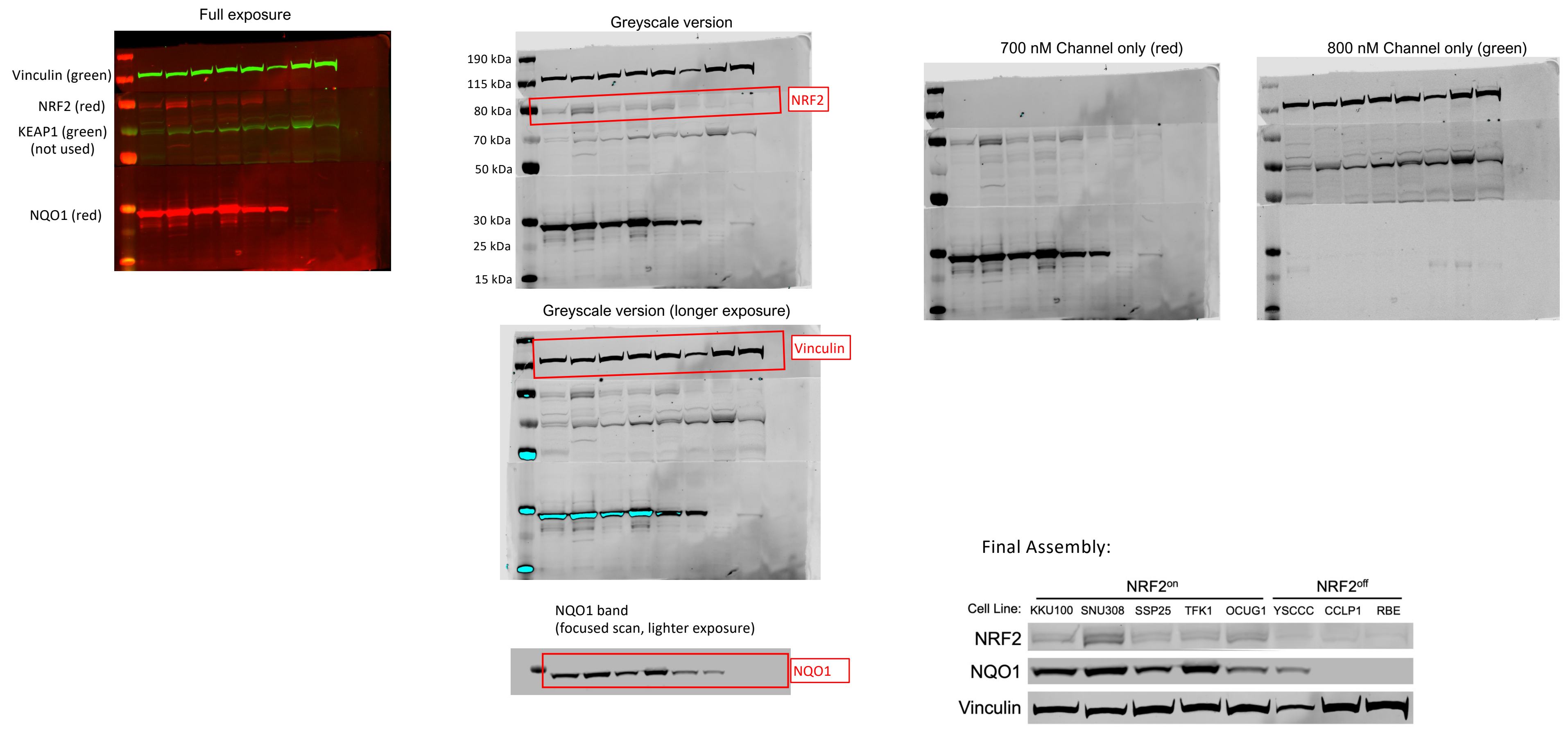
Full exposure
Vinculin (green)
NRF2 (red)
KEAP1 (green)
(not used)
NQO1 (red)
Greyscale version
190 kDa
115 kDa
80 kDa
70 kDa
50 kDa
30 kDa
25 kDa
15 kDa
NRF2
Greyscale version (longer exposure)
Vinculin
NQO1 band
(focused scan, lighter exposure)
NQO1
700 nM Channel only (red)
800 nM Channel only (green)
Final Assembly:
NRF2on
NRF2off
Cell Line:
KKU100
SNU308
SSP25
TFK1
OCUG1
YSCCC
CCLP1
RBE
NRF2
NQO1
Vinculin

Figure 1A

Full exposures

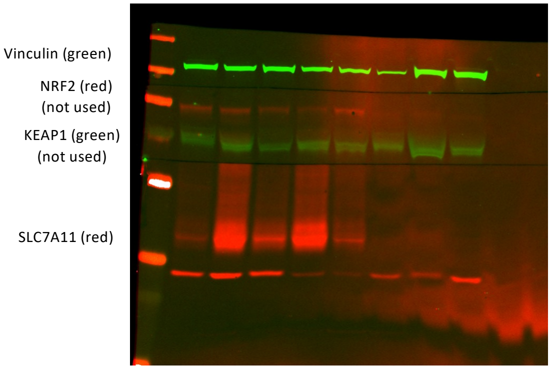

700 nM Channel only,
higher exposure (red)

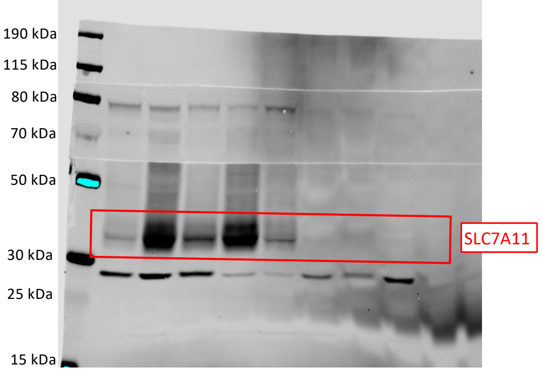

Greyscale version

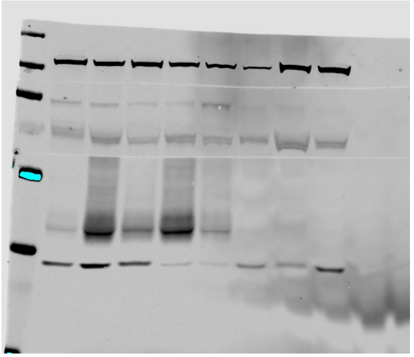

800 nM Channel only,
higher exposure (green)

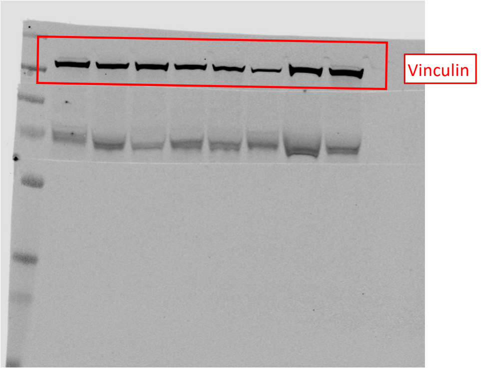

Final Assembly:

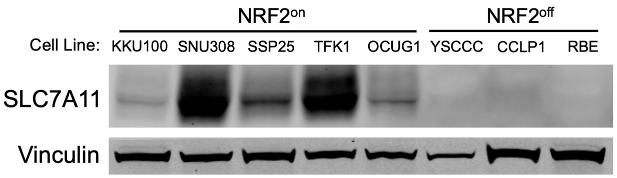

Figure 1C

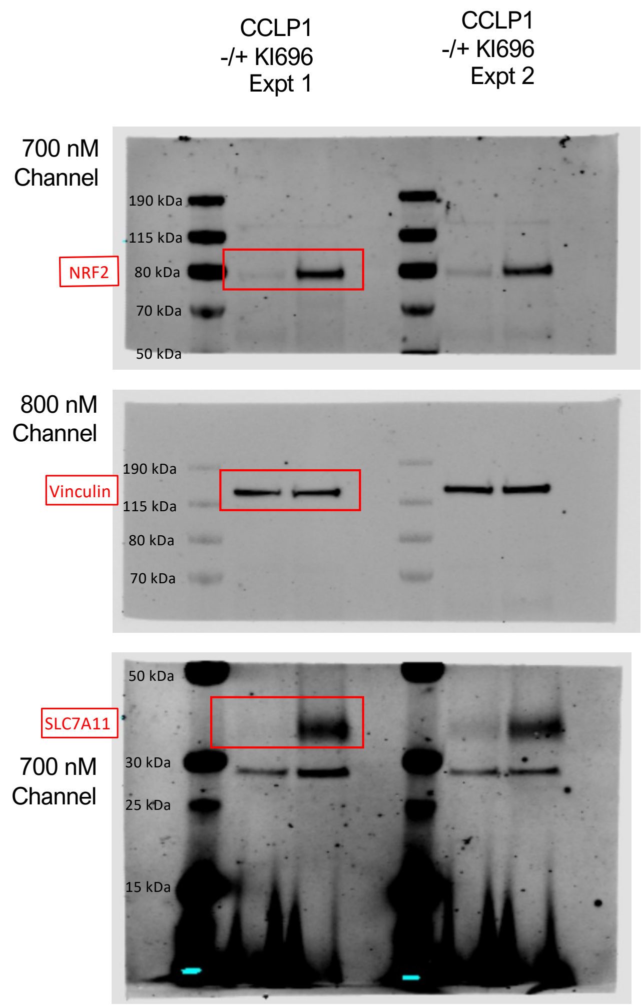

Final Assembly:

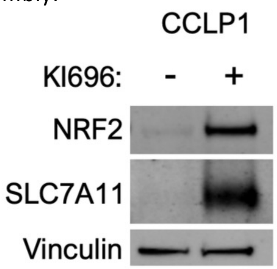

Extended Data Figure 7F

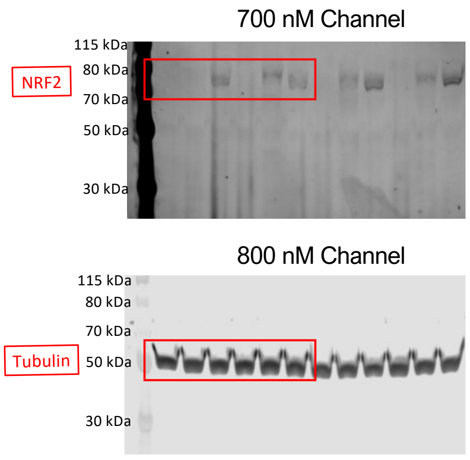

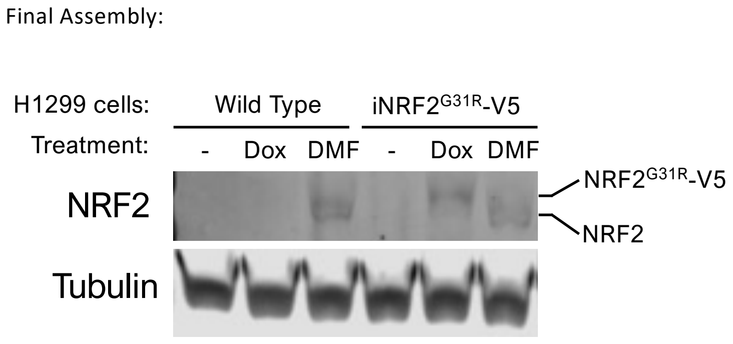

Extended Data Figure 7H

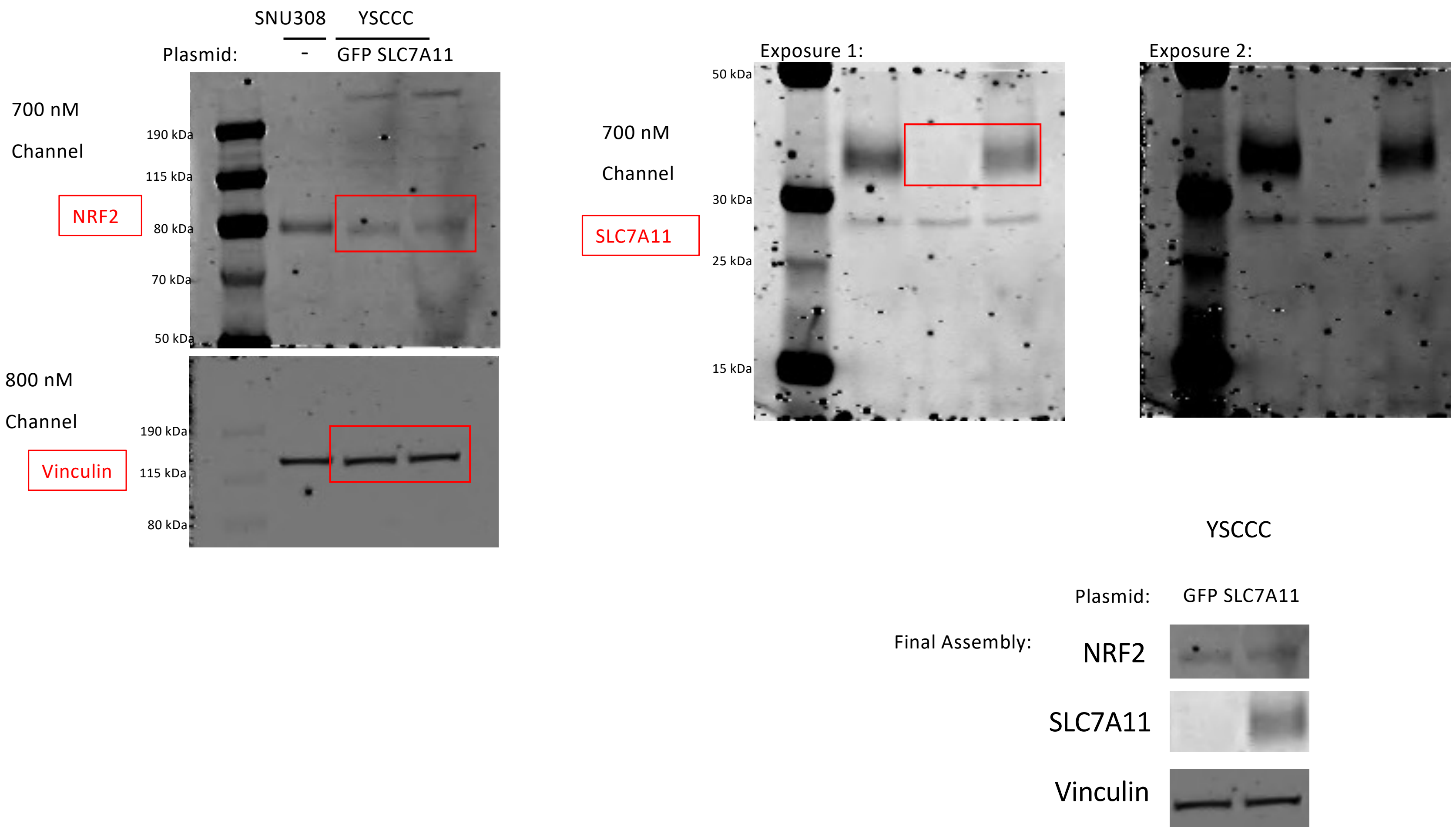

Extended Data Figure 7J
